# CAV2 Modulates Cetuximab Sensitivity in HNSCC via Ubiquitin-Mediated Disruption of the PACT-PKR Axis

**DOI:** 10.3390/cancers18071148

**Published:** 2026-04-02

**Authors:** Yun Wang, Yafei Wang, Dongqi Yuan, Shenge Liu, Peng Chen

**Affiliations:** 1 Department of Thoracic Oncology, Lung Cancer Diagnosis and Treatment Center, Tianjin Medical University Cancer Institute and Hospital, Tianjin 300060, China; wangyunyun5566@tmu.edu.cn (Y.W.); 15530171617@163.com (D.Y.); liuse1987@163.com (S.L.); 2 Department of Tumor Cell Biology, Tianjin Medical University Cancer Institute and Hospital, Tianjin 300060, China; 3 Tianjin Medical University Cancer Institute and Hospital, National Clinical Research Center for Cancer, Tianjin 300060, China; 4 State Key Laboratory of Druggability Evaluation and Systematic Translational Medicine, Key Laboratory of Cancer Prevention and Therapy, Tianjin’s Clinical Research Center for Cancer, Tianjin Medical University Cancer Institute and Hospital, Tianjin 300060, China; 5 Department of Diagnostic and Therapeutic Ultrasonography, Tianjin Medical University Cancer Institute and Hospital, Tianjin 300060, China; wangyafei@tmu.edu.cn; 6 Department of Medical Oncology, The Third Hospital of Chengde, Chengde 067000, China

**Keywords:** Caveolin-2, Cetuximab, resistance, head and neck squamous cell carcinoma, PKR, PACT

## Abstract

**Background/Objectives:** Head and neck squamous cell carcinoma (HNSCC) often exhibits limited clinical response to targeted therapies, such as Cetuximab. Identifying key drivers of tumor progression and elucidating the factors that modulate therapeutic sensitivity are essential for improving clinical outcomes. In this study, we aimed to investigate the role of CAV2 in HNSCC proliferation and its impact on Cetuximab sensitivity. **Methods:** Prognosis-associated genes in HNSCC were screened using the The Cancer Genome Atlas (TCGA) database. The functional role of Caveolin-2 (CAV2) in cell proliferation and apoptosis was assessed via Cell Counting Kit-8 (CCK-8), colony formation, and flow cytometry assays. Mechanistic insights were obtained through co-immunoprecipitation, ubiquitination assays, and proteomic analysis. The impact of CAV2 on Cetuximab sensitivity was evaluated both in vitro and in a xenograft mouse model. **Results:** Clinical analysis of 43 pairs of HNSCC tumor and adjacent normal tissues revealed that elevated CAV2 expression was significantly associated with poor prognosis in HNSCC patients (95%CI: 1.197–1.7518, *p* = 1.33 × 10^−13^). In vitro, knockdown of CAV2 suppressed cell proliferation and significantly increased apoptosis rates (from 5.1% to 10.8%, *p* = 0.004). Mechanistically, CAV2 interacted with the PACT protein and disrupted the PACT-PKR axis via the ubiquitin–proteasome pathway. Notably, CAV2 deficiency synergized with Cetuximab treatment, reducing the the half maximal inhibitory concentration (IC_50_) value by 6-fold compared with control cells and suppressing tumor growth by 48.41% in xenograft models compared to Cetuximab monotherapy (*p* < 0.0001). **Conclusions:** In conclusion, these findings establish CAV2 as a critical regulator of HNSCC progression and Cetuximab sensitivity via post-translational modulation of the PACT–PKR axis. Targeting the CAV2/PACT/PKR axis may therefore represent a promising therapeutic strategy to potentiate the efficacy of EGFR-targeted therapy in patients with HNSCC.

## Simple Summary

Head and neck squamous cell carcinoma remains a challenging malignancy with limited therapeutic options. Although the targeted therapy Cetuximab is approved for clinical use, its efficacy is often compromised in the clinic. In this study, we investigated the molecular mechanisms underlying Cetuximab sensitivity and identified CAV2 as a key modulator of this process. Our results demonstrate that elevated CAV2 levels promote tumor cell survival and render cancer cells less responsive to Cetuximab. Importantly, we show that targeting CAV2 significantly enhances drug sensitivity. These findings reveal a previously unrecognized mechanism regulating therapeutic response and suggest that combining CAV2 inhibition with existing therapies could improve outcomes for patients with head and neck cancer.

## 1. Introduction

Head and neck squamous cell carcinoma (HNSCC) represents a significant global health burden, with tongue squamous cell carcinoma (TSCC) emerging as one of its most aggressive and clinically challenging subtypes [1]. TSCC is characterized by a constellation of distinct biological features: early local invasion into the intricate musculature of the tongue, a high propensity for bilateral cervical lymph node metastasis (particularly to levels I–III) [2], and an increasing incidence among younger patients, frequently independent of Human papillomavirus (HPV) infection [3]. These anatomical and molecular attributes collectively contribute to poor clinical outcomes [4]. Moreover, TSCC tumors frequently exhibit intrinsic resistance to conventional radiotherapy and chemotherapy. For patients with recurrent or metastatic disease, the prognosis remains dismal, with limited therapeutic options beyond first-line regimens [5]. Consequently, there is an urgent need to elucidate the molecular drivers underlying TSCC pathogenesis and to identify novel targets for more effective therapeutic strategies.

To identify key oncogenic drivers in HNSCC, we interrogated The Cancer Genome Atlas (TCGA) database for prognosis-associated genes and identified Caveolin-2 (CAV2) as a top candidate. CAV2 is a core structural component of caveolae—plasma membrane invaginations abundantly expressed in epithelial cells [6,7]. While elevated CAV2 expression has been linked to poor prognosis in malignancies such as lung and pancreatic cancer [8,9,10], its functional role in HNSCC remained unexplored, prompting our investigation. Furthermore, cellular stress responses are often modulated by the double-stranded RNA-dependent protein kinase (PKR) and its primary activator, PACT [11,12]. The PACT–PKR signaling axis is frequently dysregulated in cancer and plays a critical role in determining cell fate under stress [13]. Additionally, while over 90% of HNSCC patients exhibit elevated epidermal growth factor receptor (EGFR) expression [14], the clinical efficacy of the EGFR-targeted antibody Cetuximab is often constrained by heterogeneous therapeutic responses [15]. Identifying factors that modulate the sensitivity of HNSCC cells to EGFR inhibition is therefore of great clinical importance.

To address the clinical challenge of Cetuximab resistance in HNSCC, we first integrated genomic data from the TCGA database to identify key molecular drivers associated with patient prognosis. Among the top candidates, CAV2 emerged as a potential regulator of tumor progression. In this study, our primary objective was to investigate the functional role of CAV2 in modulating HNSCC cell survival and its specific contribution to Cetuximab sensitivity. Furthermore, we aimed to elucidate the underlying biochemical mechanisms, particularly how CAV2 interacts with the PACT–PKR tumor suppressor axis via the ubiquitin–proteasome pathway. By integrating in vitro molecular assays with in vivo xenograft models, we sought to define the therapeutic potential of targeting CAV2 to overcome resistance and improve clinical outcomes for HNSCC patients.

## 2. Materials and Methods

### 2.1. Human Head and Neck Squamous Cell Carcinoma Tissue Samples

In the study, samples were gathered from patients undergoing surgical removal of HNSCC at the Department of Maxillofacial and Otorhinolaryngology Oncology and Department of Head and Neck Oncology, Cancer Hospital of Tianjin Medical University from December 2023–December 2024. The tumor samples from all patients were pathologically identified as HNSCC. Cancer tissues and their adjacent tissues were collected separately and frozen using liquid nitrogen rapidly. The preparation of protein extracts involved homogenizing tissues in a cold environment, followed by centrifugation at 4 °C. The supernatant was then collected for Western blotting analysis. The study was approved by the Research Ethics Committee of the Tianjin Medical University Cancer Institute and Hospital (approval no. AE2023026).

### 2.2. Animal Studies

BALB/c-nu female nude mice (4–5 weeks old) were purchased from Jiangsu GemPharmatech Co., Ltd. (Jiangsu, China). To establish subcutaneous model, approximately 1 × 10^6^ Ctrl/shCAV2 cells that had been resuspended in 100 μL of PBS were injected subcutaneously into the right flank of nude mice. Each group contained 12 mice, and the mice were sacrificed 5 weeks after injection. The tumor volume and weight of the mice were observed every week for 5 weeks. Tumor volume (V) was calculated using the following formula: length × width^2^ × 1/2. The mice were randomly divided into four groups when the tumor volumes reached ~150–200 mm^3^. At the experimental endpoint, mice were euthanized, and tumors were excised for further analysis.

To establish the tongue orthotopic xenograft model, SCC15-Ctrl-luci and SCC15-shCAV2-luci cells (5 × 10^5^ cells in 20 μL Matrigel, Corning, NY, USA) were carefully injected into the sublingual mucosa of 5-week-old female BALB/c nude mice using a microsyringe. Tumor engraftment was monitored by bioluminescence imaging (BLI) 7 days post-injection. Following successful tumor establishment, mice bearing SCC15-Ctrl-luci or SCC15-shCAV2-luci tumors were separately randomized into two treatment groups (*n* = 6 per group) using the RANDBETWEEN function in Microsoft Excel to generate random allocation sequences: (1) placebo group, receiving PBS via intraperitoneal injection; and (2) Cetuximab group, receiving 0.25 mg per mouse of Cetuximab (approximately 12.5 mg/kg, based on a body weight of 20 g) via intraperitoneal injection every three days (q3d) for three consecutive weeks (total of 7 doses). Additionally, tumor growth was longitudinally assessed by bioluminescence imaging (BLI) following intraperitoneal injection of D-luciferin (150 mg/kg) at indicated time points. and tumor volume was measured using calipers at indicated time points. Animals that died during the observation period were excluded from the final analysis. A total of 6 mice per group were included in the statistical analysis. At the experimental endpoint, mice were euthanized, and tongue tumors were excised for further analysis. Data were analyzed using GraphPad Prism 9.0. Two-way ANOVA followed by Tukey’s post hoc test was used for multiple comparisons.

All mice were given a regular chow diet and water and accommodated under specific pathogen-free conditions in a standard laboratory environment (21 ± 2 °C, 12 h light/dark cycle). All experimental procedures in this study were approved by the Animal Ethical and Welfare Committee of Tianjin Medical University Cancer Institute and Hospital (approval No: PMIS-2024-031) and maintained under specific-pathogen-free conditions.

### 2.3. Cell Culture, Chemicals, and Reagents

In this study, the human HNSCC cell lines SCC15 and SCC25 were acquired from the American Type Culture Collection (ATCC, Manassas, VA, USA) and supplied by the Department of Tumor Cell Biology at the Cancer Research Institute, Tianjin Cancer Hospital. The cell lines were grown in DMEM/F-12 medium (Corning, NY, USA) with an addition of 10% fetal bovine serum (FBS; PAN-Biotech, Aidenbach, Germany) and 1% penicillin/streptomycin (Hyclone, Logan, UT, USA). The HEK293T cell line, derived from human embryonic kidney cells and sourced from the American Type Culture Collection, was cultured in DMEM (Corning, NY, USA) with the addition of 10% FBS and 1% penicillin/streptomycin. A humidified atmosphere with 5% CO_2_ was used to incubate all cell lines at 37 °C.

### 2.4. CAV2 Expression Variability and Prognostic Analysis

Public RNA-seq data for head and neck squamous cell carcinoma patients downloaded from the TCGA (https://portal.gdc.cancer.gov/, accessed on 1 April 2026) database. The head and neck squamous cell carcinoma prognostic data are derived from a Cell article (LIU, Jianfang, et al., 2018, https://doi.org/10.1016/j.cell.2018.02.052) and clinicopathological characteristics are shown in Supplementary Table S2.

### 2.5. Stable Transfected Cell Lines Establishment

Lentiviral vectors encoding non-targeting control (Ctrl), shPKR, and shPACT were obtained from GeneChem (Shanghai, China). To create lentiviral particles, HEK293T cells were co-transfected with the respective transfer plasmid (shCtrl, shCAV2, shPKR, or shPACT) and the packaging plasmids VSVG and δR using polyethylenimine (PEI; Polysciences, Warrington, PA, USA). Viral supernatants were obtained 48 h following transfection. Target cells were infected using polybrene (Solarbio, Beijing, China) and selected with 1 μg/mL puromycin (Gibco, MA, USA) for a duration of 5 days to establish stable knockdown pools. The efficiency of the knockdown was verified using Western blotting and qRT-PCR.

### 2.6. Western Blotting and Antibodies

Proteins were isolated using SDS-PAGE at 80–140 V and then transferred onto Immobilon-P membranes from Millipore, MA, USA. The membranes were blocked with 5% non-fat milk or 3% BSA in TBST for an hour at room temperature before being incubated with primary antibodies overnight at 4 °C. The day after, membranes were incubated with HRP-conjugated secondary antibodies for one hour at room temperature, and signals were detected using an ECL kit by Pierce, IL, USA.

The following antibodies were employed: CAV2 (1:1000, NBP1-31116, Novus Biologicals, Centennial, CO, USA), PKR (1:1000, 12297S, CST, Danvers, MA, USA), PACT (1:1000, 13490S, CST, Danvers, MA, USA), Ki67 (62548SF, 1:1000, CST, Danvers, MA, USA), Ubiquitin (3936S, 1:1000, CST, Danvers, MA, USA), PARP (9532S, 1:1000, CST, Danvers, MA, USA), HRP-conjugated Goat Anti-Rabbit IgG (1:4000, SA00001-2, Proteintech, Wuhan, China), and HRP-conjugated Goat Anti-Mouse IgG (1:4000, SA00001-1, Proteintech, Wuhan, China). The antibody information used for Western Blot is shown in the Supplementary Table S4.

### 2.7. Colony Formation Assays

Cells were placed in 6-well plates at a concentration of 800 cells per well and grown for 10 to 14 days. The formed cell colonies were rinsed three times with PBS, fixed with 4% paraformaldehyde for 30 min, and stained with 0.5% crystal violet (Solarbio, Beijing, China). Using a digital imaging system, images of the colonies were taken. The experiments were all done in sets of three.

### 2.8. EdU Assay

The BeyoClick™ EdU Cell Proliferation Kit with Alexa Fluor 594 (Beyotime, Shanghai, China, C00788L) was utilized to assess cell proliferation. Cells were seeded in 48-well plates at a density of 1 × 10^4^ cells per well and cultured for 24 h. The medium was then supplemented with 10 μM EdU, followed by incubation for 2 h at 37 °C under 5% CO_2_.

Cells were washed with PBS, fixed with 4% paraformaldehyde for 30 min at room temperature, and permeabilized with 0.3% Triton X-100 for 15 min after the EdU-containing medium was removed. To visualize incorporated EdU, the Click reaction was carried out following the manufacturer’s guidelines. DAPI was used to counterstain the nuclei for 10 min, and images were captured with an inverted fluorescence microscope equipped with a digital camera. Three distinct replicates were carried out for each condition in the experiment.

### 2.9. Cell Viability Assay

Cells were placed in 96-well plates at a density of 1000 cells per well in 100 μL of culture medium. At each time of measurement, 10 μL of CCK-8 solution (Dojindo Laboratories, Kumamoto, Japan) was added to each well, followed by incubation at 37 °C for 2–4 h. A microplate reader was used to measure the optical density at 450 nm. Cell proliferation curves were generated from measurements taken continuously over a period of 4–5 days. Each experiment was conducted with a minimum of three independent replicates. The CCK-8 absorbance at 450 nm was adjusted by removing the blank control and then normalized to Day 0, where the fold change is set to 1. Cell growth was determined by dividing the corrected optical density on any given day by the corrected optical density on Day 0.

### 2.10. RNA Extraction and qRT-PCR

Total RNA extraction and qRT-PCR assays were performed as previously described [16]. The relative mRNA expression was calculated using the $2^{-\Delta \Delta Ct\;}$ method [17]. The primers used are listed in the Supplementary Table S5.

### 2.11. IHC

The Tianjin Medical University Cancer Institute and Hospital provided tissue sections embedded in paraffin. These sections were deparaffinized with xylene and rehydrated through a graded series of ethanol. Citrate buffer (pH = 6.0) was used for antigen retrieval at 95 °C for 15 min, followed by blocking endogenous peroxidase activity with 3% hydrogen peroxide at room temperature for 15 min. The sections were incubated overnight at 4 °C with primary antibodies targeting CAV2 (1:100, Novus, NBP1-31116) or Ki67 (1:200, CST, 62548SF) diluted in an antibody diluent. The sections were incubated with an HRP-conjugated secondary antibody (PV-6001 kit, Zhongshan Biotechnology, Zhongshan, China) for one hour at 37 °C after washing. For signal detection, a DAB substrate kit (Zhongshan Biotechnology) was used, followed by counterstaining with hematoxylin. Images were acquired using a brightfield microscope (Olympus BX61, Tokyo, Japan) with different objective lenses.

### 2.12. TUNEL Assay

Cells were placed on sterile coverslips in 12-well plates a day before the experiment. After incubation, the cells were treated with 4% paraformaldehyde at room temperature for 30 min and then permeabilized using 0.3% Triton X-100 for 10 min at room temperature. Following the manufacturer’s instructions, apoptotic cells were detected using a TUNEL assay kit by applying 50 μL of TUNEL reaction mixture to each sample and incubating at 37 °C for one hour in the dark. The nuclei were counterstained with DAPI for 10 min after PBS washing. Coverslips were mounted with an anti-fade medium and examined under a fluorescence microscope.

### 2.13. Co-Immunoprecipitation (Co-IP)

A commercial kit (PK10008, Proteintech, Wuhan, China) was used to perform Co-IP assays according to the manufacturer’s instructions, with minor adjustments. The adherent cells were washed three times with pre-chilled PBS and lysed in IP lysis buffer. The lysates were spun at 12,000× *g* for 10 min at 4 °C to clear them. Equal protein lysate amounts were incubated overnight at 4 °C with either the target antibody or a species-matched IgG control. Protein A/G beads were subsequently added and incubated for 2 h at 4 °C with gentle rotation. The beads were washed 4–5 times with 800 μL of wash buffer, and the proteins that were bound were eluted using an elution buffer. The samples were then neutralized, combined with 5× SDS loading buffer, and heated at 95 °C for 5 min.

### 2.14. Flow Cytometry Analysis

Cell apoptosis was examined using the PE Annexin V Apoptosis Detection Kit (BD Pharmingen™, Franklin Lakes, NJ, USA, 559763). After washing with chilled PBS, cells were resuspended in 1× Binding Buffer at a concentration of 1 × 10^6^ cells/mL. 100 μL cell suspension was placed into a flow cytometry tube and mixed with 5 μL of PE Annexin V and 5 μL of 7-AAD, then left to incubate for 15 min at room temperature in the dark. After incubation, 400 μL of 1× Binding Buffer was added to each tube. Samples were subjected to flow cytometry using a BD FACS instrument within an hour. The analysis distinguished between viable cells (Annexin V^−^/7-AAD^−^), early apoptotic cells (Annexin V^+^/7-AAD^−^), late apoptotic cells (Annexin V^+^/7-AAD^+^), and necrotic cells (Annexin V^−^/7-AAD^+^).

### 2.15. Apoptosis Enzyme-Linked Immunosorbent Assay (ELISA)

Using the Cellular DNA Fragmentation ELISA kit (Roche, Basel, Switzerland 11585045001), apoptosis was quantitatively assessed according to the manufacturer’s instructions. Cells were seeded in 96-well plates at a density of 1 × 10^4^ cells per well and pre-labeled with BrdU for 24 h. Cells were treated with the apoptosis inducer CCCP (10 μM) and incubated for 1–6 h at 37 °C. After incubation, the cells were collected by centrifuging at 300× *g* for 10 min, and the supernatant was gently removed. Cell pellets were lysed with the incubation buffer supplied for 30 min at room temperature, then centrifuged at 800× *g* for 10 min to collect the supernatant with fragmented DNA. The supernatant underwent the usual ELISA process to measure DNA fragmentation. Absorbance was recorded at 450 nm with a microplate reader, and the results were compared to untreated controls. Three independent experiments were performed in triplicate.

### 2.16. Proximity Ligation Assay

The Duolink^®^ PLA was conducted on paraffin-embedded HNSCC tissue sections from the Department of Maxillofacial, Ear, Nose and Throat Oncology at Tianjin Medical University Cancer Hospital to identify protein–protein interactions in situ. Sections were deparaffinized and rehydrated, then antigen retrieval was performed using a sodium citrate buffer with a pH of 6.0. The Duolink^®^ InSitu Red Starter Kit Mouse/Rabbit (Sigma-Aldrich, St. Louis, MO, USA) was utilized as per the manufacturer’s guidelines. The primary antibodies used were CAV2, PKR (1:100, 18244-1-AP, Rabbit, Proteintech, Wuhan, China or 1:100, sc-6282, mouse, Santa Cruz, Dallas, TX, USA), and PACT (1:100, sc-377103, mouse, Santa Cruz, Dallas, TX, USA), and they were incubated overnight at 4 °C in a humidified chamber. After washing, the steps of ligation and amplification were performed as directed. Nuclei were counterstained with a mounting medium containing DAPI from the kit. Images were captured using a fluorescence microscope. Three independent experiments were performed with appropriate controls.

### 2.17. Ubiquitination Assay

For the ubiquitination assay, SCC15 and SCC25 cells were transiently transfected with the HA-Ub plasmid together with either control shRNA or sh-CAV2 for 36 h. To inhibit proteasomal degradation of ubiquitinated proteins, cells were incubated with MG-132 (10 μM) for 8 h prior to harvesting. Cells were lysed in denaturing lysis buffer (1% SDS, 150 mM NaCl, 10 mM Tris-HCl, pH = 8.0) and boiled at 95 °C for 10 min to disrupt non-covalent protein–protein interactions. The lysates were then diluted 1: 10 in PBS and subjected to immunoprecipitation using an anti-PKR/PACT antibody or a normal rabbit IgG (as a negative control). The precipitated complexes were analyzed by Western blotting. The PKR/PACT polyubiquitination levels were detected using an anti-ubiquitin antibody.

### 2.18. Quantitative Proteomics Analysis (IP-MS)

SCC15-Ctrl and SCC15-shCAV2 cell lines lysates were subjected to immunoprecipitation using anti-CAV2 antibodies. The precipitated protein complexes were separated by SDS-PAGE and stained with Coomassie brilliant blue. The gel lanes were excised, decolored, and digested with sequencing-grade trypsin at 37 °C overnight. The resulting peptides were extracted, desalted, and analyzed using a RIGOL L-3000 High-Performance Liquid Chromatography (HPLC) system.

The quantitative proteomics analysis was performed by Beijing Qinglian Biotech Co., Ltd. (Beijing, China). Briefly, peptide samples were separated using a RIGOL L-3000 HPLC system (Rigol Technologies, Beijing, China) and subsequently analyzed on an Orbitrap Fusion mass spectrometer (Thermo Fisher Scientific, Waltham, MA, USA). Data acquisition was conducted in a data-dependent acquisition (DDA) mode with a mass resolution of 120,000 for full MS scans. The specific elution gradient and instrument parameters followed the standard protocols provided by the service provider.

### 2.19. Statistical Analysis

Differences between the two groups were assessed using Student’s *t*-test, and the Wilcoxon rank sum test was utilized for analyzing expression differences. The prognostic outcomes were analyzed using the univariate Kaplan–Meier method and the multivariate Cox proportional hazards model, with data evaluation performed using R version 4.2.1. Western blot band intensities were quantified using ImageJ (version 1.54r, National Institutes of Health, Bethesda, MD, USA; https://imagej.nih.gov/ij/, accessed on 1 April 2026). Data were analyzed using GraphPad Prism 9.0 (GraphPad Software, San Diego, CA, USA). Differences were deemed statistically significant if the *p*-value was below 0.05.

## 3. Results

### 3.1. CAV2 Is Overexpressed in HNSCC and Correlates with Poor Patient Prognosis

**Figure 1:**
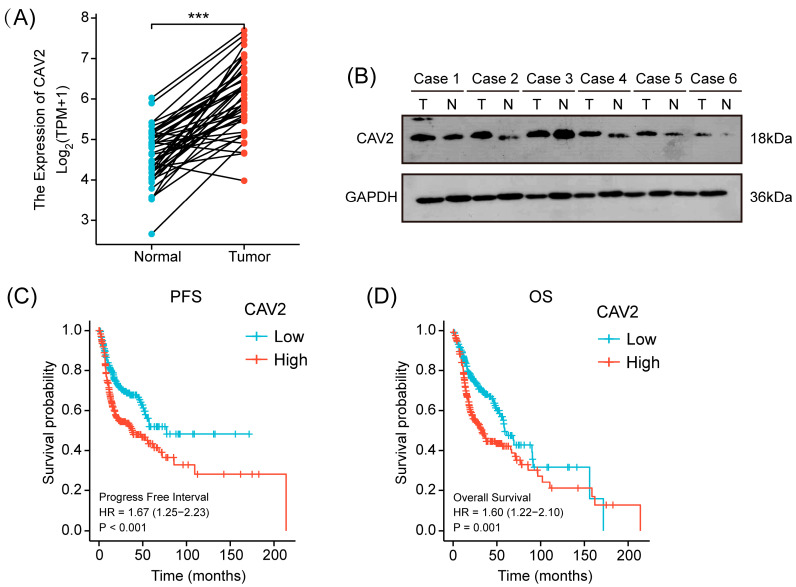
CAV2 is overexpressed in HNSCC and correlates with poor patient prognosis. (**A**). The TCGA dataset analysis reveals CAV2 mRNA expression in tumor and corresponding normal tissues across 43 HNSCC (T) pairs and adjacent non-tumor tissues (N). (**B**). Western blot was used to analyze CAV2 in five pairs of human HNSCC tumors and nearby non-cancerous tissues, with GAPDH serving as a loading control. (**C**,**D**). Kaplan–Meier survival curves based on TCGA dataset showing progress-free survival (**C**) and overall survival (**D**) of HNSCC patients stratified by high vs. low CAV2 expression. Log-rank test was used for statistical comparison. *** *p* < 0.001. Original western blots are presented in File S1.

To explore the clinical implications of CAV2 in HNSCC, we began investigating the TCGA database. CAV2 expression was found to be significantly higher in HNSCC tissues than in normal controls, according to tumor transcriptome analysis (Figure 1A). We examined 6 pairs of tumor and adjacent non-tumor tissues from 50 patients diagnosed with HNSCC at Tianjin Cancer Hospital and Institute. The baseline clinical characteristics of these patients are summarized in Supplementary Table S1. As shown in Figure 1B, while CAV2 protein levels were significantly upregulated in most HNSCC cases, Case 3 showed no obvious increase. This variation likely reflects the inherent inter-patient heterogeneity common in HNSCC, where individual molecular profiles can be influenced by tumor stage, histological grade, or specific genetic backgrounds. Despite this individual variation, the overall trend across the entire cohort remained statistically significant.

Critically, elevated CAV2 expression was strongly associated with adverse patient outcomes. Patients with elevated CAV2 levels had notably shorter overall and progression-free interval compared to those with lower CAV2 expression (Figure 1C,D). Notably, this is highly consistent with the protein-level clinical validation previously reported by our research group [16]. In our prior study, IHC analysis of clinical HNSCC patient tissues confirmed that CAV2 protein is markedly overexpressed in tumors and serves as a strong predictor of poor clinical prognosis.

### 3.2. CAV2 Promotes HNSCC Cell Proliferation In Vitro and In Vivo

**Figure 2:**
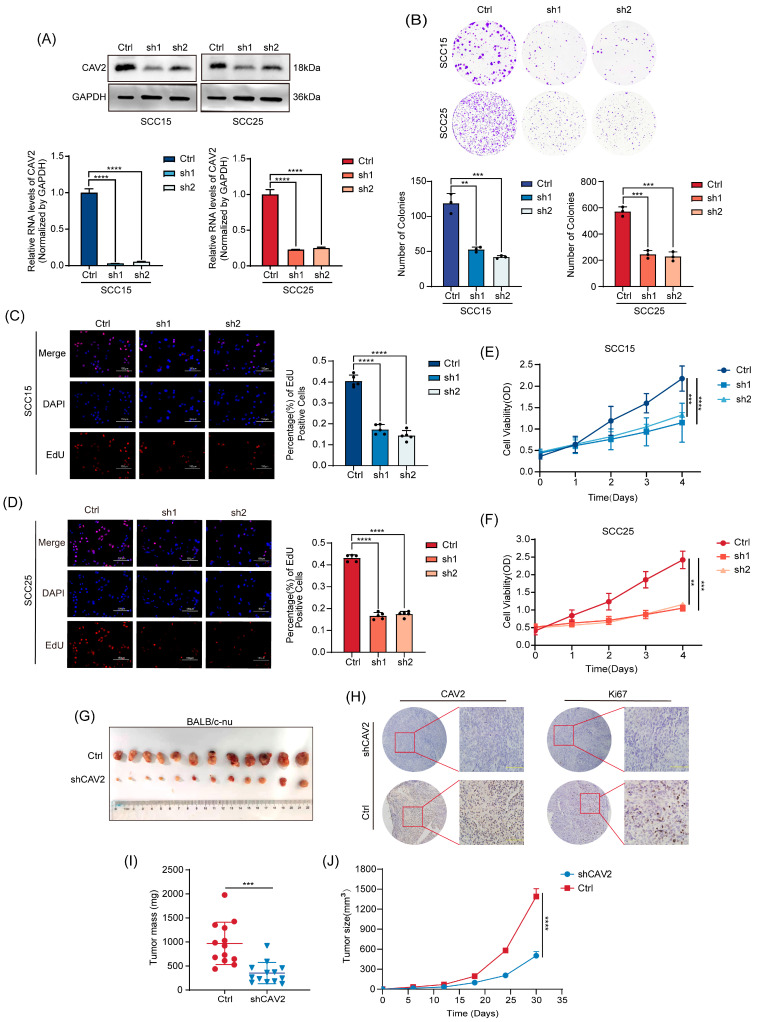
CAV2 promotes HNSCC cell proliferation in vitro and in vivo. (**A**). The infection of SCC15 and SCC25 cells with lentivirus carrying shRNA against CAV2 was performed. Knockdown efficiency was confirmed by Western blotting for proteins and qRT-PCR for mRNA, with GAPDH as the loading control. (**B**) Colony formation assay and (**C**,**D**) EdU staining were performed to assess proliferative capacity in control and CAV2-knockdown SCC15 (**C**) and SCC25 (**D**) cell lines. Scale bar, 50 μm. The bar diagram represents the fraction of EdU-positive cells. (**E**,**F**). Cell growth curves of SCC15 (**E**) and SCC25 (**F**) cells following CAV2 knockdown. The Y-axis shows the fold change in cell count compared to Day 0. The data are expressed as mean ± SEM from three separate experiments. (**G**). Images representing subcutaneous xenograft tumors from control and CAV2-knockdown cells. (**H**). Immunohistochemical staining of CAV2 and the proliferation marker Ki67 in tumor sections. Scale bar, 100 μm. (**I**). Tumor size was tracked from day 6 following inoculation, and the tumor’s weight was documented at the endpoint (**J**). Data are presented as mean ± SEM; *n* = 13 mice per group. ** *p* < 0.01, *** *p* < 0.001, **** *p* < 0.0001; ns, not significant. Original western blots are presented in File S1.

To analyze the function of CAV2 in HNSCC, we created stable CAV2-knockdown (shCAV2) and control (Ctrl) cells in the SCC15 and SCC25 lines, verifying transfection efficiency through Western blot and qRT-PCR. (Figure 2A). A series of in vitro functional assays demonstrated that CAV2 depletion significantly impaired malignant phenotypes. Specifically, colony formation and EdU staining assays revealed a marked reduction in proliferative capacity (Figure 2B–D), while CCK-8 assays confirmed a decrease in overall cell viability (Figure 2E,F). To confirm these results in a living organism, we implanted the engineered cells under the skin of nude mice. As expected from the in vitro data, knocking down CAV2 led to a significant decrease in tumor size and weight (Figure 2G,I,J). IHC analysis of the xenograft tissues further supported the conclusion that CAV2 drives tumor growth (Figure 2H). Collectively, these results from complementary models establish that CAV2 is a critical promoter of HNSCC proliferation.

### 3.3. CAV2 Suppresses Apoptosis in HNSCC Cells

**Figure 3:**
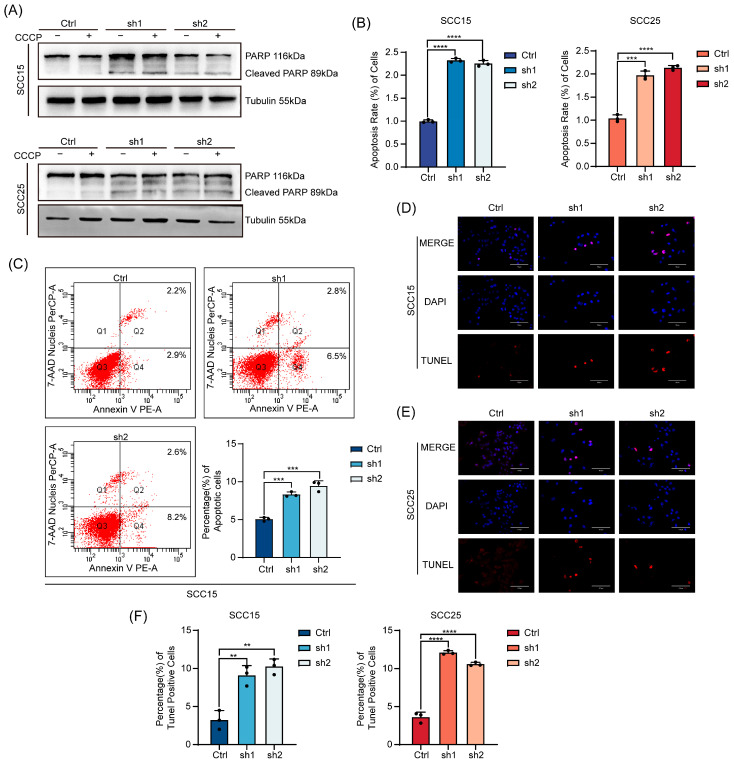
CAV2 suppresses apoptosis in HNSCC cells. (**A**). Western blot was used to analyze cleaved PARP protein levels in SCC15 and SCC25 cells with CAV2 knockdown and control, both with and without CCCP treatment. GAPDH served as the loading control. (**B**). DNA fragmentation in the cell supernatant was measured by ELISA in control and CAV2-knockdown groups.in SCC15 and SCC25 cells. (**C**). Representative images of Flow cytometry in control and CAV2-knockdown SCC15. (**D**,**E**). Representative images of TUNEL staining in control and CAV2-knockdown SCC15 (**D**) and SCC25 (**E**) cell lines. Scale bar, 50 μm. (**F**). Quantification of TUNEL-positive cells from multiple fields. Data are presented as mean ± SEM from three independent experiments. ** *p* < 0.01, *** *p* < 0.001, **** *p* < 0.0001; ns, not significant. Original western blots are presented in File S1.

Given that tumorigenesis is propelled by both uncontrolled proliferation and evasion of cell death [18]. Apoptosis is the main and most extensively researched type of programmed cell death [19], so we explored if CAV2 plays a role in inhibiting apoptosis, we next investigated whether CAV2 contributes to apoptosis suppression. A panel of complementary assays consistently demonstrated that CAV2 knockdown potently induces apoptosis. Immunoblot analysis showed a marked increase in cleaved PARP—a hallmark of apoptosis—upon CAV2 silencing (Figure 3A). Notably, the addition of the apoptosis inducer CCCP did not further elevate cleaved PARP levels, suggesting that CAV2 knockdown alone may maximally activate the apoptotic pathway under the tested conditions. Correspondingly, ELISA quantification revealed a significant increase in DNA fragmentation, a key biochemical marker of apoptosis [20], in the supernatant of CAV2-knockdown cells (Figure 3B). The total apoptotic effect was directly confirmed by flow cytometry (Annexin V^+^/PI^−^ and Annexin V^+^/PI^+^ cells), which quantified a higher percentage of apoptotic cells following CAV2 depletion (Figure 3C). Finally, TUNEL staining provided visual evidence, showing a greater abundance of TUNEL-positive (red-stained) apoptotic cells in CAV2-knockdown cultures compared to controls (Figure 3D–F). In conclusion, CAV2 functions as a critical suppressor of apoptosis in HNSCC cells.

### 3.4. CAV2 Interacts with the PACT-PKR Axis but Does Not Function as a Scaffold

**Figure 4:**
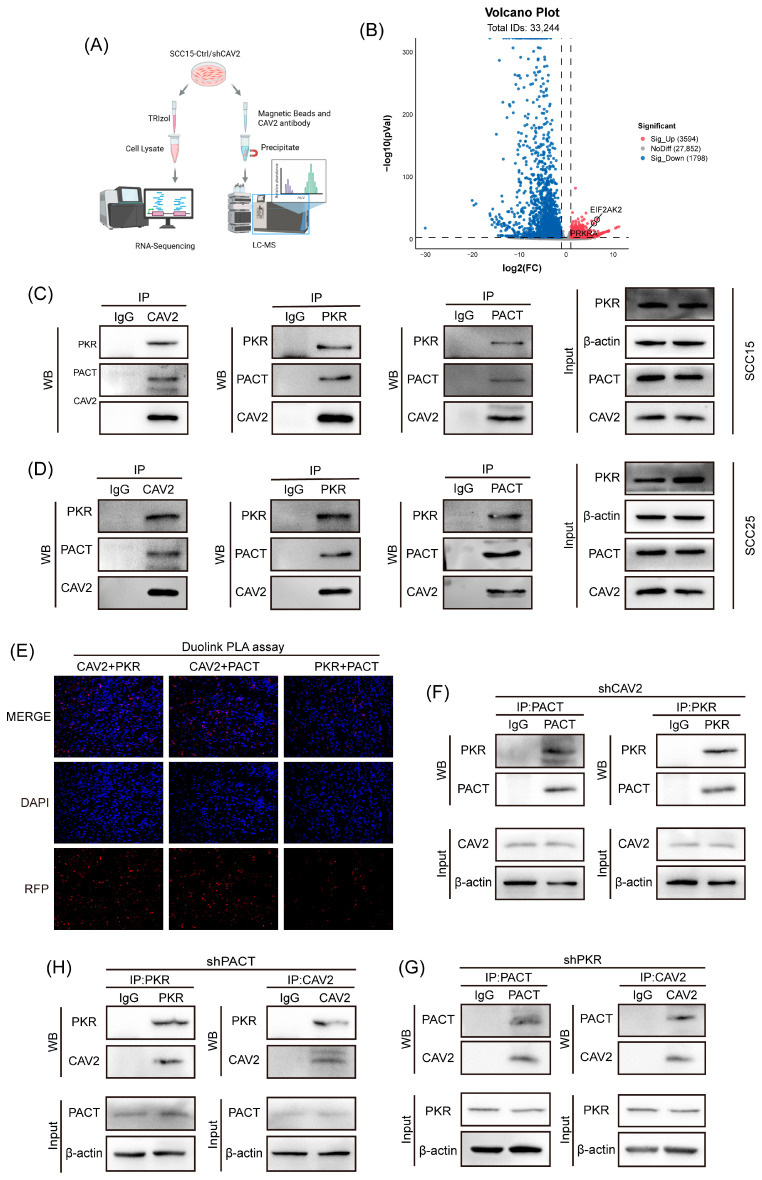
CAV2 interacts with the PACT-PKR axis but does not function as a scaffold. (**A**). The workflow integrates transcriptomic profiling (RNA-seq) and TMT-based quantitative proteomics to pinpoint high-confidence effector molecules. (**B**). Volcano plot displaying differentially expressed proteins following CAV2 silencing. Significantly upregulated (Red) and downregulated (Blue) proteins are highlighted (|Log_2_FoldChange| ≥ 2, *p* < 0.001). (**C**,**D**). Co-immunoprecipitation (Co-IP) assays in SCC15 (**C**) and SCC25 (**D**) cells using antibodies against CAV2, PKR, and PACT, followed by immunoblotting with the indicated antibodies. Total cell lysates (Input) and IgG controls are shown. (**E**). Duo-link PLA was performed on human HNSCC tissue sections to detect protein–protein interactions between CAV2, PKR, and PACT. Red fluorescent dots represent specific protein interactions. Scale bar, 50 μm. (**F**). Co-IP of endogenous PKR and PACT in control and CAV2-knockdown SCC15 cells. (**G**). Co-IP of CAV2 and PACT in control and PKR-knockdown SCC15 cells. (**H**). Co-IP of CAV2 and PKR in control and PACT-knockdown SCC15 cells. All Co-IPs were performed with the indicated antibodies and analyzed by Western blotting. Original western blots are presented in File S1.

To systematically elucidate the molecular mechanism by which CAV2 promotes HNSCC progression and Cetuximab resistance, we performed an integrated multi-omics screening strategy (Figure 4A). Initially, transcriptomic sequencing (RNA-seq) was conducted comparing SCC15-Ctrl and SCC15-shCAV2 cells. Using stringent criteria (|Log_2_FoldChange| ≥ 2, *p* < 0.001) we identified 5392 differentially expressed genes (DEGs), with PRKRA and EIF2AK2 prominently distributed in the up-regulated region of the volcano plot (Figure 4B). Simultaneously, TMT-based quantitative proteomics was employed to profile the proteomic landscape, identifying 1020 proteins in total (Supplementary Table S3).

To narrow down the most high-confidence candidates, we focused on binary expression patterns in the proteomics data—specifically, proteins that were baseline-undetectable in the SCC15-Ctrl group (Not/Peak found) but were significantly restored upon CAV2 knockdown (*n* = 139). By intersecting these 139 proteins with the RNA-seq DEGs (|Log_2_FoldChange| ≥ 3) we identified 7 consensus candidates via Venn diagram analysis (Supplementary Figure S1A).

Among these 7 targets, PRKRA (encoding PACT) and its downstream kinase EIF2AK2 (encoding PKR) emerged as the most significant hits within the stress-response and tumor-suppressor axis. The |Log_2_FoldChange| values from the transcriptome further confirmed the robust induction of the PACT-PKR axis following CAV2 depletion (Supplementary Figure S1B). These results suggest that CAV2 may exert its oncogenic function by suppressing the PACT-PKR tumor suppressor axis at both the transcriptional and translational levels.

**Figure 5:**
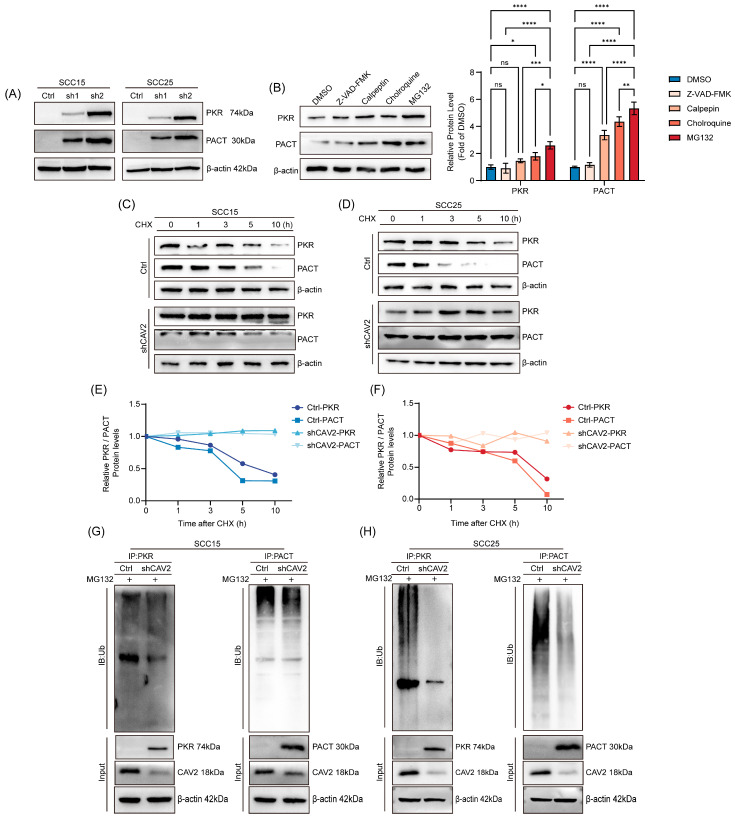
CAV2 promotes the ubiquitin-mediated proteasomal degradation of PKR and PACT. (**A**). Western blot analysis of PKR and PACT protein levels in control and CAV2-knockdown SCC15 and SCC25 cells. GAPDH served as a loading control. (**B**). (Left Panel) Immunoblot analysis of PKR and PACT expression in control and CAV2-knockdown cells treated with various protease inhibitors: MG-132 (proteasome inhibitor), chloroquine (lysosome inhibitor), calpeptin (calpain inhibitor), and Z-VAD-FMK (caspase inhibitor). Cells were treated with 10μM of each inhibitor for 24 h. (Right Panel) Quantitative densitometric analysis of PKR and PACT protein levels from the immunoblots. The relative protein expression was normalized to DMSO. Data are presented as the mean ± SD of three independent experiments. Statistical significance was determined using two-way ANOVA. (**C**–**F**). (**C**,**D**) Western blot analysis and (**E**,**F**) quantification of PKR and PACT protein stability in control and CAV2-knockdown cells treated with 100 μg/mL cycloheximide (CHX) for the indicated time periods. Protein bands were quantified using ImageJ software (version 1.54r, National Institutes of Health, Bethesda, MD, USA) and normalized to GAPDH. Data are presented as mean ± SEM from three independent experiments. (**G**,**H**). Immunoblot analysis of polyubiquitinated PKR and PACT in control and CAV2-knockdown SCC15 and SCC25 cells. Cells were treated with 10 μM MG-132 for 24 h prior to immunoprecipitation with anti-PKR or anti-PACT antibodies, followed by Western blotting with anti-ubiquitin antibody. * *p* < 0.05, ** *p* < 0.01, *** *p* < 0.001, **** *p* < 0.0001; ns, not significant. Original western blots are presented in File S1.

Given the established role of PKR dysregulation in tumorigenesis and therapeutic response [21,22,23], we prioritized PKR for further validation. Immunoblotting confirmed that CAV2 knockdown significantly upregulated both PKR and its key activator, PACT (Figure 5A). To definitively confirm the specificity of the interaction, we performed reciprocal Co-IP assays in both SCC15 and SCC25 cells. As shown in Figure 4C,D, CAV2 specifically co-precipitated with PKR, and conversely, PKR successfully pulled down CAV2. Furthermore, to stringently validate their spatial colocalization and direct physical interaction in situ, we performed a proximity ligation assay (PLA) in tissue sections (Figure 4E), which yielded specific positive signals, further substantiating the specific CAV2–PKR/PACT binding. Based on our previous finding that CAV2 functions as a scaffold protein [16], we hypothesized that CAV2 might facilitate the PKR–PACT interaction. However, Co-IP assays revealed that CAV2 knockdown did not impair the endogenous binding between PKR and PACT (Figure 4F). Moreover, PKR knockdown did not reduce the CAV2–PACT interaction (Figure 4G), nor did PACT knockdown affect CAV2–PKR binding (Figure 4H). This interaction pattern indicates that CAV2 associates independently with PKR and PACT and does not serve as a scaffold for their complex formation.

### 3.5. CAV2 Promotes the Ubiquitin-Mediated Proteasomal Degradation of PKR and PACT

To elucidate the mechanism by which CAV2 regulates PKR and PACT protein levels, we first noted that their mRNA levels were unaltered upon CAV2 knockdown (Supplementary Figure S2A,B), suggesting post-translational regulation. To identify the predominant degradation pathway, we employed inhibitors targeting major proteolytic systems. While mild increases in PKR and PACT protein levels were observed following treatment with various proteolytic inhibitors, the most substantial and robust accumulation was triggered by the proteasome inhibitor MG-132, compared to inhibitors of lysosomal (chloroquine), calpain (calpeptin), or caspase (Z-VAD-FMK) activities (Figure 5B). This indicates that the ubiquitin–proteasome pathway is the predominant mechanism responsible for the basal turnover of PKR and PACT in these cells.

We subsequently investigated whether CAV2 actively modulates the degradation machinery of the PACT-PKR axis. Cycloheximide (CHX) chase assays revealed that the turnover rate of PKR and PACT proteins was markedly accelerated in the presence of CAV2, whereas their stability was significantly restored upon CAV2 knockdown (Figure 5C–F). To further delineate the specific degradation pathway orchestrated by CAV2, we utilized pharmacological inhibitors. The CAV2-mediated reduction in PKR and PACT protein levels was completely reversed by treatment with the specific proteasome inhibitor MG-132, but not by lysosomal or caspase inhibitors (Figure 5B,G,H). Collectively, these robust functional findings provide definitive evidence that CAV2 primarily drives the degradation of the PACT-PKR tumor suppressor axis via the ubiquitin–proteasome system (UPS), thereby shutting down its downstream signaling.

### 3.6. PKR and PACT Mediate the Pro-Oncogenic Effects of CAV2 in HNSCC

**Figure 6:**
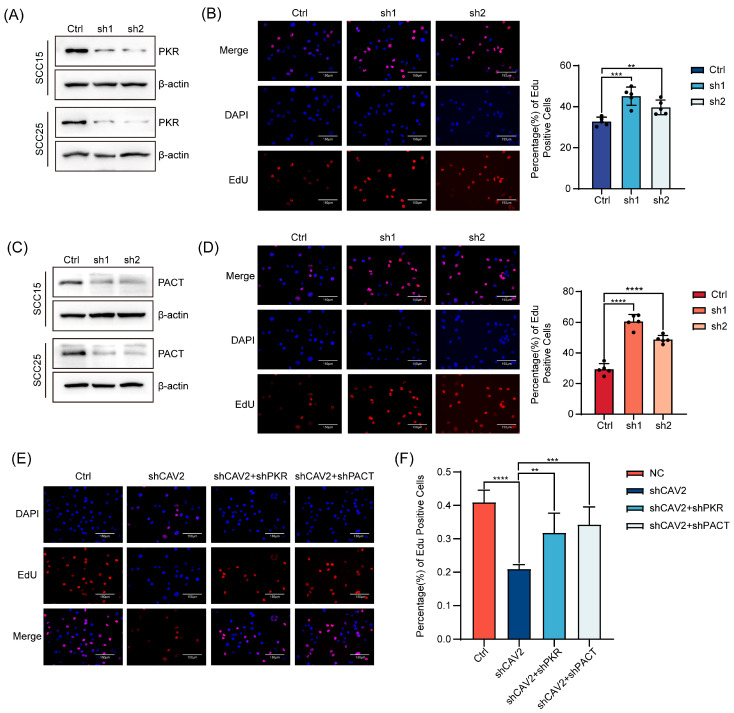
PKR and PACT mediate the pro-oncogenic effects of CAV2 in HNSCC. (**A**). Western blot analysis confirming PKR protein levels in control and PKR-knockdown SCC15 and SCC25 cells. GAPDH served as a loading control. (**B**). (Left) Representative images of EdU staining in control and PKR-knockdown cells. Scale bar, 50 μm. (Right) Quantification of EdU-positive cells. Data are presented as mean SEM from three independent experiments. (**C**). Western blot analysis confirming PACT protein levels in control and PACT-knockdown SCC15 and SCC25 cells. GAPDH served as a loading control. (**D**). (Left panel) Representative images of EdU staining in ±control and PACT-knockdown cells. Scale bar, 50 μm (Right panel). Quantification of EdU-positive cells. Data are presented as mean ± SEM from three independent experiments. (**E**). EdU assay demonstrating that the anti-proliferative effect of CAV2 knockdown was partially reversed by co-depletion of PKR or PACT. (**F**). Quantification of (**E**) EdU-positive cells. Data are presented as mean ± SEM from three independent experiments. ** *p* < 0.01, *** *p* < 0.001, **** *p* < 0.0001; ns, not significant. Original western blots are presented in File S1.

Having established that CAV2 downregulation elevates PKR and PACT protein levels (Figure 5A), we next asked whether these downstream effectors functionally contribute to the tumor-promoting phenotype of CAV2. We generated stable PKR- and PACT-knockdown HNSCC cell lines (SCC15 and SCC25), with knockdown efficiency confirmed by immunoblotting and qRT-PCR (Figure 6A,C).

Strikingly, depletion of either PKR or PACT significantly enhanced cell proliferation (Figure 6B,D), phenocopying the effect of CAV2 overexpression. This suggested that PKR and PACT act as downstream functional mediators. To test this hypothesis directly, we performed rescue experiments. Notably, co-depletion of PKR or PACT partially but significantly reversed the proliferation defect induced by CAV2 knockdown (Figure 6E,F). Collectively, these functional data position PKR and PACT as key downstream effectors through which CAV2 drives HNSCC proliferation.

To further explore the signaling cascade downstream of the PACT-PKR axis, we evaluated the phosphorylation status of eIF2α, a canonical target of PKR. We observed an upregulation of phosphorylated eIF2α following CAV2 silencing (Supplementary Figure S3A). This suggests that CAV2 depletion attenuates global protein synthesis by hyperactivating the PKR–eIF2α pathway, providing an additional mechanistic perspective on how CAV2 promotes tumor progression by bypassing translational shutoff. Furthermore, to examine the response to cellular stress, we activated endoplasmic reticulum (ER) stress using tunicamycin in PKR- or PACT-deficient cells. In these cells, tunicamycin failed to significantly suppress proliferation, underscoring the essential role of the PACT–PKR axis in mediating ER stress-induced growth inhibition in HNSCC (Supplementary Figure S3B,C).

### 3.7. CAV2 Modulates Cetuximab Sensitivity in HNSCC by Targeting the PACT-PKR Signaling Axis

**Figure 7:**
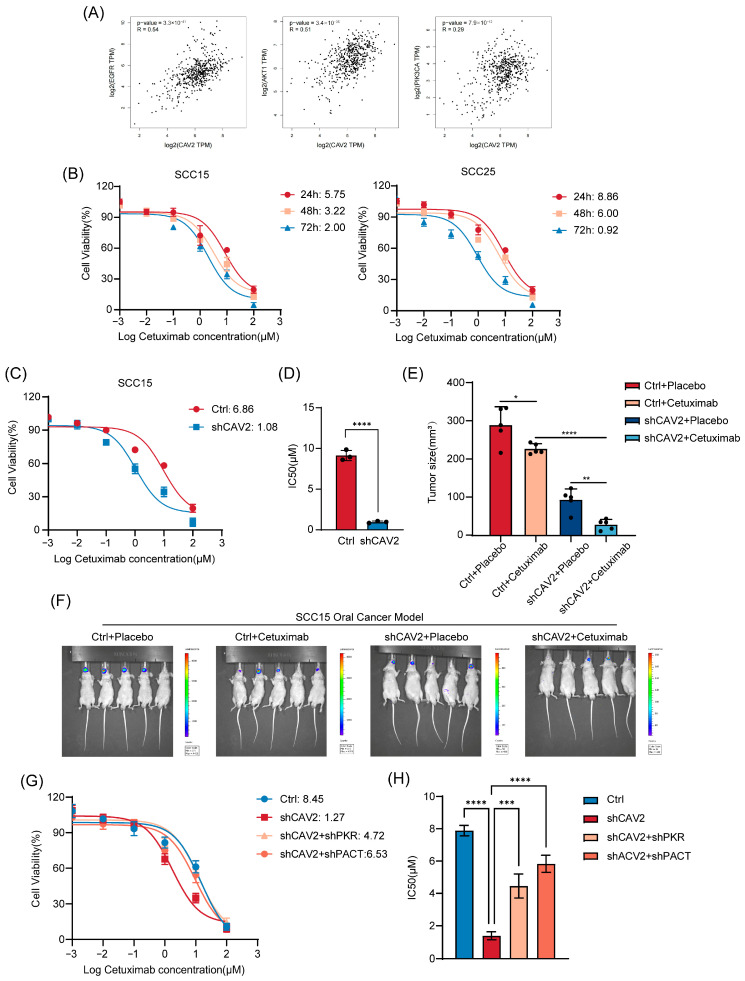
CAV2 Modulates Cetuximab Sensitivity in HNSCC by Targeting the PACT-PKR Signaling Axis. (**A**). Scatter plots showing the expression correlation of CAV2 with representative molecules of the EGFR signaling pathway from a public transcriptomic database GEPIA (Gene Expression Profiling Interactive Analysis). Pearson correlation coefficient (r) and *p*-value are indicated. (**B**). IC_50_ values of Cetuximab were determined at 24 h, 48 h, and 72 h in (Left panel) SCC15 and (Right panel) SCC25 cell lines using CCK-8 assays. (**C**,**D**). Dose–response curves of Cetuximab after 48 h treatment in SCC15-shCAV2 and SCC15-shCtrl cells (**C**). Bar graph comparing the IC_50_ values between the two groups (**D**). Data are presented as mean ± SD (*n* = 3). **** *p* < 0.0001 by unpaired Student’s *t*-test. (**E**,**F**). Representative bioluminescence images of mice bearing SCC15-Ctrl-luci or SCC15-shCAV2-luci tumors at the experimental endpoint after treatment with either PBS (control) or Cetuximab (**F**). Mice were randomly assigned to treatment groups (*n* = 5 per group). Data are presented as mean ± SD from three independent experiments. Bar graph showed the final tumor volumes measured from the four experimental groups described in (**E**). Data are presented as mean ± SEM (*n* = 5). * *p* < 0.05, ** *p* < 0.01, **** *p* < 0.0001 by one-way ANOVA with Tukey’s post hoc test. (**G**,**H**). Dose–response curves of Cetuximab after 48 h treatment in SCC15-Ctrl, SCC15-shCAV2, SCC15-shCAV2 + shPKR and shCAV2+shPACT cells (**G**). Bar graph comparing the IC_50_ values among the four groups (**H**). Data are presented as mean ± SD (*n* = 3). *** *p* < 0.001, **** *p* < 0.0001 by one-way ANOVA with Tukey’s post hoc test.

The EGFR/PI3K/AKT signaling pathway plays a fundamental role in tumorigenesis and progression in HNSCC [24]. While CAV2 has been reported to positively correlate with EGFR signaling in renal cell carcinoma [25], its relationship with this pathway in HNSCC remained unclear. Analysis of public transcriptomic databases revealed a significant positive correlation between CAV2 expression and key components of the EGFR pathway in HNSCC (Figure 7A), suggesting a potential functional interplay.

To evaluate the functional impact of this association, we first established the baseline sensitivity of HNSCC cell lines to Cetuximab, with SCC15 and SCC25 exhibiting basal IC_50_ values of 5.75 μM and 8.68 μM, respectively (Figure 7B). Notably, CAV2 knockdown dramatically sensitized these cells to Cetuximab, evidenced by a substantial reduction in IC_50_ values (Figure 7C,D). To validate these in vitro findings in a physiologically relevant context, we employed an orthotopic tongue cancer model. While Cetuximab monotherapy showed limited efficacy, its combination with CAV2 silencing (shCAV2) led to a profound reduction in tumor burden (Figure 7E,F). Crucially, the Coefficient of Drug Interaction (CDI) was calculated as 0.501, demonstrating a potent synergistic effect (CDI < 0.7) rather than a mere additive response.

Having confirmed that CAV2 depletion effectively restores Cetuximab sensitivity, we next sought to determine whether the PACT-PKR signaling axis is the primary functional mediator of this drug-resistant phenotype. Therefore, we performed pathway-specific rescue experiments by co-depleting PACT or PKR in CAV2-silenced HNSCC cells. Consistent with our earlier results, CAV2 knockdown robustly sensitized cells to Cetuximab; however, this sensitization was significantly reversed upon concurrent knockdown of either PACT or PKR, with cells regaining substantial resistance (Figure 7G,H). Cell viability assays further confirmed that the disruption of the PACT-PKR axis serves as a critical bypass mechanism that overrides the vulnerability induced by CAV2 loss.

Collectively, these functional validation and rescue data establish that CAV2 modulates Cetuximab sensitivity in HNSCC primarily by suppressing the PACT-PKR tumor suppressor axis.

## 4. Discussion

Globally, head and neck squamous cell carcinoma (HNSCC) is a significant health issue, ranking among the top common cancers. In China alone, the disease incidence and associated mortality remain alarmingly high, with tens of thousands of new cases and deaths projected annually [26]. Although the majority of patients have locally advanced disease, which is linked to worse survival rates, treatment options are still limited [27]. In particular, resistance, whether inherent or acquired, often diminishes the efficacy of EGFR-targeted treatments like Cetuximab, underscoring the urgent need to find new targets and combination strategies to improve outcomes.

Caveolin-2 (CAV2), a core structural component of caveolae, has emerged from our TCGA-based screening as a strong prognostic indicator in HNSCC [28]. CAV2 is the most strongly associated molecule with patient prognosis in head and neck squamous carcinoma that we mined from the TCGA database. Although CAV1, its well-characterized paralog, has been extensively studied in cancer biology [29,30], the functional role of CAV2 remains comparatively undefined, particularly in HNSCC.

In this study, we systematically defined the oncogenic role of CAV2 in HNSCC. Analysis of public databases and our clinical specimens consistently revealed that CAV2 is highly expressed in HNSCC tissues, and its elevated expression correlates with poor patient prognosis. Analysis of public databases along with our clinical specimens consistently demonstrated that CAV2 is prominently expressed in HNSCC tissues, and its elevated levels are connected to poor patient outcomes. Our data suggest that CAV2 may act as a critical brake on the intrinsic apoptotic pathway. Its removal unleashes a near-maximal apoptotic response, diminishing the incremental effect of additional stressors like CCCP. This phenomenon resembles the ceiling effect observed in other oncogene-depletion models, where loss of a survival factor commits cells irreversibly to death.

Proteomic profiling identified PKR as a key molecule associated with CAV2. Although PKR was initially recognized for its role in viral infection, recent studies have implicated it in diverse biological processes, including endoplasmic reticulum stress, oxidative stress, and tumorigenesis [31,32,33]. In our models, CAV2 silencing led to upregulation of PKR, which subsequently promoted apoptosis. Given that PACT is a well-established upstream activator of PKR and facilitates its function through direct binding, we initially hypothesized that CAV2, PKR, and PACT form a ternary complex, with CAV2 acting as a scaffold to modulate the PKR–PACT interaction. However, co-immunoprecipitation assays refuted this assumption, indicating that the three proteins do not form a stable complex but instead engage in pairwise interactions independent of the third partner. Since CAV2 knockdown did not alter the mRNA levels of PKR or PACT, we postulated that CAV2 regulates their expression post-transcriptionally. Using cycloheximide (CHX) chase assays, we found that the stability of both PKR and PACT was enhanced in CAV2-knockdown cells. Furthermore, among several protease pathway inhibitors, only the proteasome inhibitor MG132 caused significant accumulation of PKR and PACT. Subsequent ubiquitination immunoprecipitation confirmed that CAV2 depletion promotes polyubiquitination and proteasomal degradation of both proteins, indicating that CAV2 stabilizes PKR and PACT by inhibiting their ubiquitin-mediated degradation.

PKR has been observed to have context-dependent functions in different cancers, where it acts as an oncogene in breast cancer, melanoma, and colon cancer, yet it seems to serve as a tumor suppressor in HNSCC [9,34], consistent with our findings. In rescue experiments, concurrent knockdown of PACT or PKR partially reversed the anti-proliferative effect caused by CAV2 deletion, supporting the notion that the PACT–PKR axis operates downstream of CAV2 [35]. However, PKR was identified as a tumor suppressor in head and neck squamous cell carcinoma cells [36,37], which is consistent with our experimental results. The RESCUE experiments found that PACT-PKR knockdown could partially rescue the decline in cell malignancy caused by CAV2 deletion.

PKR suppresses tumor growth primarily by phosphorylating eIF2α, core mechanism that curtails overall protein synthesis and shifts the cellular translation program during stress [34,38,39]. Specifically, PKR-mediated phosphorylation of eIF2α shuts down the assembly of the eIF2 complex, thereby inhibiting the initiation of translation for the majority of cellular mRNAs [35]. In our study, phosphorylated eIF2α (p-eIF2α) levels were significantly upregulated upon CAV2 silencing (Supplementary Figure S3A), suggesting that the depletion of CAV2 relieves the suppression of the PKR pathway and effectively re-activates core growth-inhibitory signaling. Furthermore, given that the PKR-eIF2α axis is central to the ER stress response [40], we found that CAV2 knockdown concurrently triggers both PACT–PKR activation and the upregulation of ER stress markers. While the precise causal hierarchy between these two molecular events in HNSCC warrants further investigation, existing literature supports the notion that PKR activation modulates proteostatic pathways and contributes to heightened cellular stress sensitivity [31,41].

A pivotal finding of our study is that CAV2-mediated Cetuximab resistance is driven by the proteasomal degradation of the PACT-PKR tumor suppressor axis. To further elucidate the underlying biochemical mechanism governing this turnover, we performed a proteome-wide interaction prediction using UbiBrowser 2.0, a high-performance platform for E3-substrate bioinformatics [42]. Remarkably, SMURF1 (SMAD Specific E3 Ubiquitin Protein Ligase 1) was identified as a top-ranked, high-confidence E3 ligase candidate for PKR (Score = 0.785, Confidence Level: High).

SMURF1 is a member of the HECT domain E3 ligase family, originally identified for its role in transforming growth factor-β (TGF-β) signaling through SMAD degradation [43]. Subsequent studies have expanded its substrate spectrum to various non-SMAD signaling proteins, including RhoA and other cytoplasmic transducers, thereby dictating cell fate and motility [44,45]. Crucially, clinical evidence demonstrates that SMURF1 is significantly overexpressed in HNSCC cohorts, acting as a potent oncogenic driver that promotes tumor aggressiveness and predicts poor patient survival [46].

Given that CAV2 frequently functions as a scaffolding protein or spatial organizer within the plasma membrane and cytoplasm, we propose a mechanistic model in which overexpressed CAV2 facilitates the spatial recruitment of SMURF1 into close proximity with the PACT-PKR complex. This CAV2-dependent “scaffolding effect” likely triggers the proximity-induced ubiquitination and subsequent degradation of the PKR axis, ultimately enabling HNSCC cells to bypass the growth-inhibitory stress response typically induced by Cetuximab. Although the direct physical interaction between SMURF1 and the PACT-PKR axis warrants further biochemical validation, our findings—supported by both computational modeling and established literature—provide a novel molecular link between caveolin-mediated protein scaffolding and E3-dependent protein triage in head and neck cancer therapy resistance. Ultimately, our study demonstrates that restoring this stress-responsive signaling via CAV2 depletion significantly sensitizes HNSCC cells to Cetuximab-induced apoptosis, offering a potential therapeutic vulnerability for overcoming EGFR-targeted therapy resistance.

The majority of HNSCC tumors have high levels of EGFR expression and activate the EGFR/PI3K/AKT pathway [14], although Cetuximab, an EGFR monoclonal antibody, is FDA-approved for HNSCC treatment, its clinical efficacy is often limited. In our orthotopic xenograft model, CAV2-deficient tumors showed enhanced sensitivity to Cetuximab compared with controls. This finding positions CAV2 as a potential target for combination therapy, which may help improve the therapeutic response to Cetuximab in HNSCC patients. More importantly, our findings demonstrate that CAV2 depletion improves Cetuximab sensitivity not by directly altering canonical EGFR receptor dependency, but by restoring the ubiquitin-protected PACT-PKR stress response pathway, which ultimately drives the resistant cells into apoptosis under Cetuximab treatment.

Our findings are consistent with and extend recent studies on HNSCC progression and drug resistance. MRPL21 has been found to promote cisplatin resistance in HNSCC by activating the PI3K/AKT/mTOR pathway and blocking autophagy [47]. Similarly, mutations in the FAT1 gene have been found to promote resistance to immune checkpoint inhibitors by shaping an immunosuppressive tumor microenvironment [48]. These studies collectively suggest that the malignant progression and therapeutic resistance of HNSCC involve the coordinated dysregulation of multiple signaling pathways. Our work positions CAV2 and its regulated PACT–PKR axis within this complex molecular network, providing a novel perspective for understanding HNSCC pathogenesis.

It should be noted that while our results demonstrate that CAV2 expression modulates the sensitivity of HNSCC cells to Cetuximab, this study has several limitations. Firstly, our in vitro experiments were primarily conducted using parental HNSCC cell lines SCC15 and SCC25 to evaluate baseline sensitivity; further investigations utilizing specific acquired Cetuximab-resistant models are warranted to fully elucidate the role of CAV2 in the maintenance of resistance. Secondly, while our initial attempts to investigate this axis in non-TSCC cell lines (such as FaDu) were hindered by severe lentiviral toxicity and poor post-transfection cell viability, our comprehensive evaluation of pan-HNSCC clinical cohorts confirms that CAV2 overexpression consistently dictates poor prognosis across various anatomical sub-sites. This clinical evidence strongly supports the broad applicability of the CAV2/PACT-PKR paradigm beyond TSCC. Furthermore, as Cetuximab resistance is highly multifactorial, potential cross-talk between CAV2 and other resistance bypass pathways, such as alternative receptor tyrosine kinase activation, warrants further investigation. Finally, while our prognostic analyses were supported by the TCGA database, prospective studies involving large-scale, multi-center clinical cohorts are still imperative to definitively validate the clinical utility of CAV2 as a predictive biomarker for Cetuximab efficacy in HNSCC patients.

## 5. Conclusions

In summary, our study identifies CAV2 as a critical modulator of Cetuximab resistance in HNSCC by mediating the ubiquitin-dependent degradation of the PACT-PKR tumor suppressor axis. Our results demonstrate that knockdown of CAV2 restores the sensitivity by approximately 84.26%. Mechanistically, CAV2 significantly reduces the protein half-life of PACT and PKR by promoting their polyubiquitination, resulting in a 60–70% decrease in their steady-state protein levels compared to the control group. Furthermore, in vivo xenograft models confirmed that targeting the CAV2-mediated pathway combined with Cetuximab achieved a significantly higher tumor inhibition rate, 71.43%, compared to Cetuximab monotherapy, 23.02% (*p* < 0.0001). These objective outcomes highlight the potential of the CAV2/PACT/PKR axis as a diagnostic biomarker and a therapeutic target for overcoming EGFR-targeted therapy resistance in patients with head and neck cancer.

## Data Availability

The original contributions presented in the study are available under request to correspondence authors.

## Supplementary Materials

The following supporting information can be downloaded at: https://www.mdpi.com/article/10.3390/cancers18071148/s1. Figure S1: Integrated Multi-omics Screening for CAV2 Downstream Targets. Figure S2: Effects of CAV2 knockdown on the transcript levels of PKR and PACT. Figure S3: CAV2 knockdown attenuates eIF2α phosphorylation and PKR/PACT knockdown rescues TM-induced proliferation inhibition. Table S1: Clinicopathological characteristics of 50 HNSCC patients in Tianjin Cancer Hospital and Institute. Table S2: Clinicopathological characteristics of TCGA-HNSCC cohort (Liu et al., Cell, 2018). Table S3: The comprehensive list of the 1020 identified proteins of TMT proteomics. Table S4: The antibody information used for Western blot is shown in the table below. Table S5: The primer sequences used for qRT–PCR analyses are shown in the table below. File S1: Original western blots.

## Abbreviations

The following abbreviations are used in this manuscript:

CAV2	Caveolins 2
Co-IP	Co-immunoprecipitation (Co-IP)
ELISA	Enzyme-linked Immunosorbent Assay
HNSCC	Head and Neck Squamous Cell Carcinoma
IHC	Immunohistochemistry
OS	Overall Survival
PFS	Profression-free Survival
PLA	Proximity Ligation Assay
TCGA	The Cancer Genome Atlas
TSCC	Tongue Squamous Cell Carcinoma
