# Supplementary material for: CAV2 Modulates Cetuximab Sensitivity in HNSCC via Ubiquitin-Mediated Disruption of the PACT-PKR Axis
Source: Cancers (Basel). 2026 Apr 2;18(7):1148. doi: 10.3390/cancers18071148 (PMC13072382; doi:10.3390/cancers18071148)
Supplement: Supplementary file 1 [file cancers-18-01148-s001.zip › cancers-4203275_Supplementary_Materials.docx]

**Supplementary Materials:**

**Supplementary Figure S1.** Integrated Multi-omics Screening for CAV2 Downstream Targets.


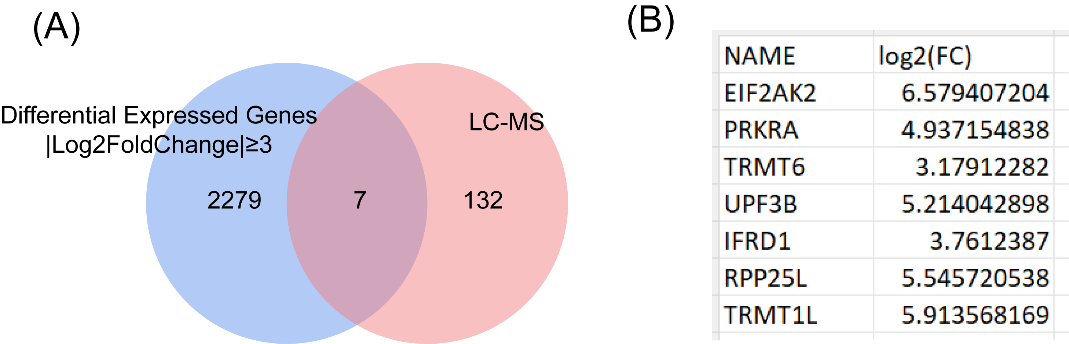


**Supplementary Figure S1.** Integrated Multi-omics Screening for CAV2 Downstream Targets. **(A)** Venn diagram illustrating the intersection between RNA-seq DEGs (|Log_2_FoldChange|≥3) and TMT-identified proteins that were specifically restored in the shCAV2 group (defined as proteins not detected in Ctrl cells but quantified in shCAV2 cells, n=139). **(B)** The intersection yielded 7 candidate genes, including PRKRA and EIF2AK2, which were selected for further mechanistic validation.

**Supplementary Figure S2.** Effects of CAV2 knockdown on the transcript levels of PKR and PACT.


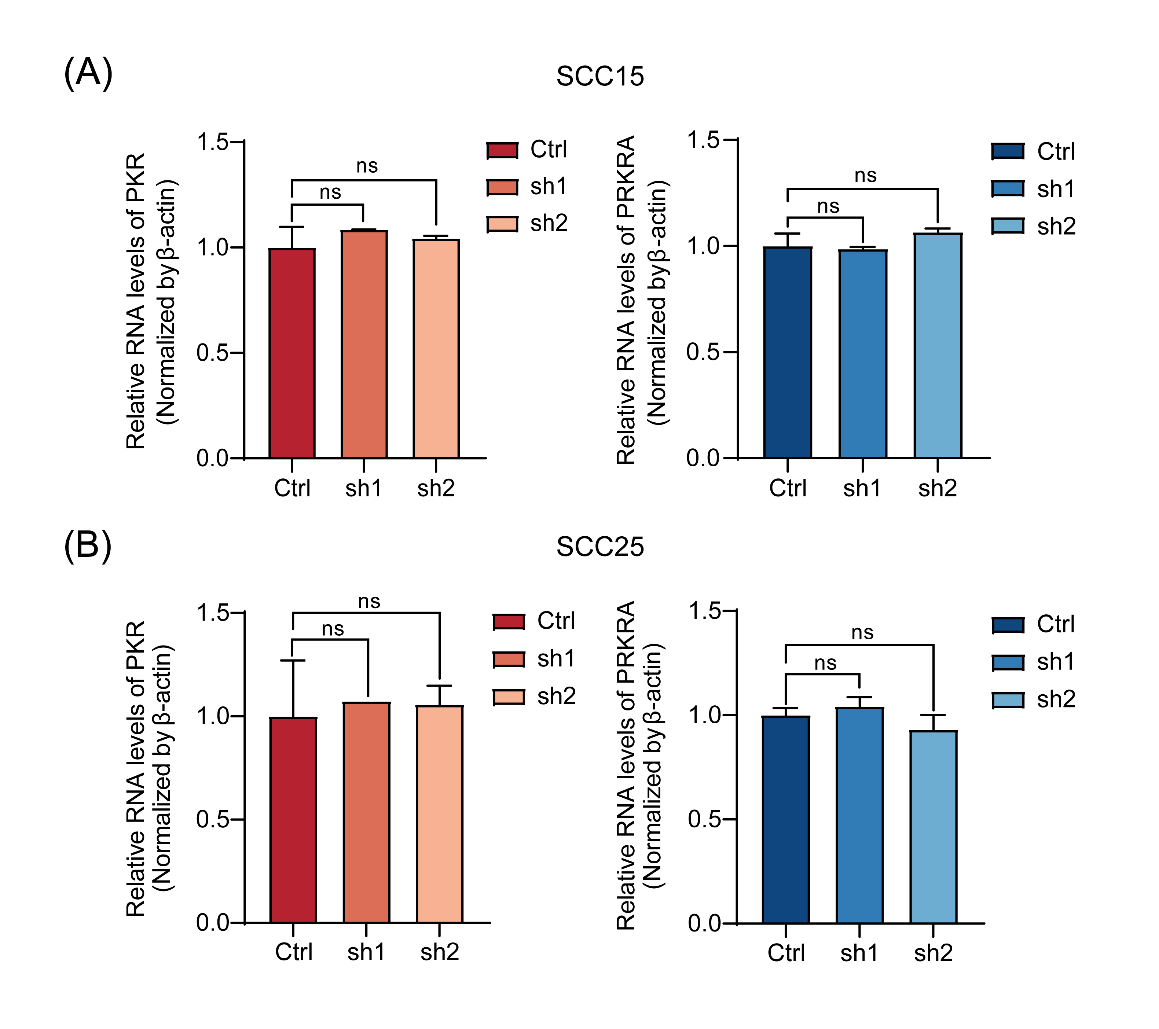


**Supplementary Figure S2**. Effects of CAV2 knockdown on the transcript levels of PKR and PACT in (A) SCC15 and (B) SCC25 cells, determined by qRT-PCR. All data are shown as the mean ± SEM from three independent experiments. ns, not significant.

**Supplementary Figure S3.** CAV2 knockdown attenuates eIF2α phosphorylation and PKR/PACT knockdown rescues TM-induced proliferation inhibition.


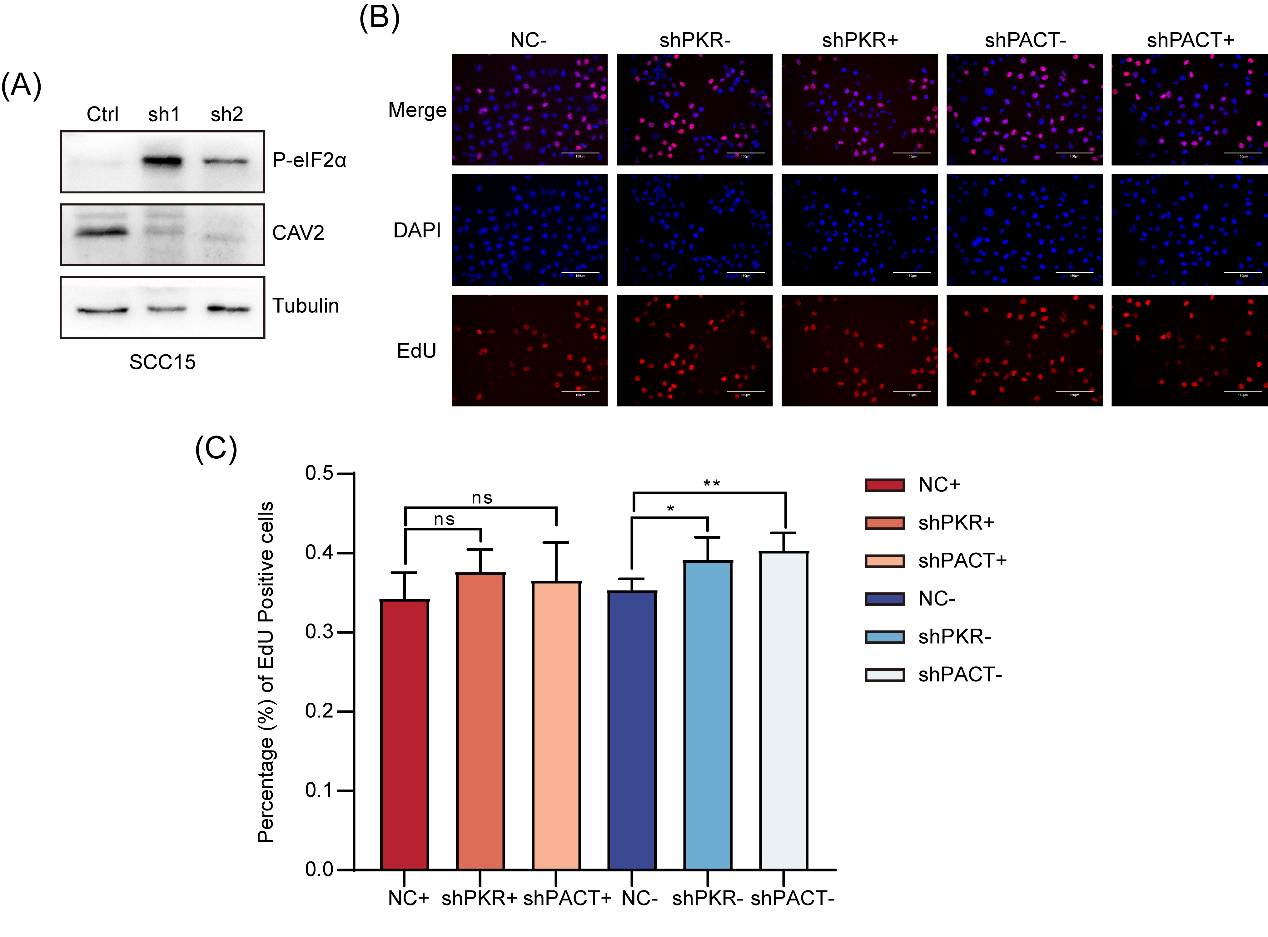


**Supplementary Figure S3.** CAV2 knockdown attenuates eIF2α phosphorylation and PKR/PACT knockdown rescues TM-induced proliferation inhibition. **(A).** Western blot analysis of phosphorylated eIF2α (p-eIF2α) protein levels in control and CAV2-knockdown SCC15 and SCC25 cells. GAPDH served as a loading control. **(B)**. Representative images (Left panel) and quantitative analysis (Right panel) of EdU staining in control, PKR-knockdown, and PACT-knockdown cells treated with 100μg/mL tunicamycin (TM, an ER stress inducer) for 24 hours. Scale bar, 50μm. **(C)**. Statistical quantification of the data shown in (B). Data are presented as the mean ± SD from three independent experiments. Data are presented as mean ± SEM from three independent experiments. * *p* < 0.05, ** *p* < 0.01 by one-way ANOVA with Tukey's post-hoc test.

| **Supplementary Table S1.** Clinicopathological characteristics of 50 HNSCC patients in Tianjin Cancer Hospital and Institute. | |
| --- | --- |
| **Characteristics** | **Number of cases (%)** |
| **Age** |  |
| ≥60 | 28(56.0) |
| <60 | 22(44.0) |
| **Gender** |  |
| Male | 36(72.0) |
| Female | 13(28.0) |
| **Anatomic subdivision** |  |
| Tonsil | 4(8.0) |
| Oral tongue | 12(24.0) |
| Larynx | 11(22.0) |
| Oral cavity | 7(14.0) |
| Floor of mouth | 6(12.0) |
| Alveolar Ridge | 2(4.0) |
| Base of tongue | 3(6.0) |
| Buccal Mucosa | 2(4.0) |
| Oropharynx | 1(2.0) |
| Hard Palate | 0(0.0) |
| Hypopharynx | 1(2.0) |
| Lip | 1(2.0) |
| **Alcohol history** |  |
| Yes | 33(66.0) |
| No | 17(34.0) |
| **Tobacco smoking history** |  |
| No | 11(22.0) |
| Yes | 39(78.0) |
| **Histologic grade** | 48 |
| G1 | 6(12.5) |
| G2 | 28(58.3) |
| G3 | 12(25.0) |
| G4 | 2(4.2) |
| **Clinical T stage** |  |
| T1 | 3(6.0) |
| T2 | 14(28.0) |
| T3 | 15(30.0) |
| T4 | 18(36.0) |
| **Clinical N stage** |  |
| N0 | 23(46.0) |
| N1 | 8(16.0) |
| N2 | 15(30.0) |
| N3 | 4(8.0) |
| **Clinical stage** |  |
| I | 2(4.0) |
| II | 10(20.0) |
| III | 11(22.0) |
| IV | 27(54.0) |

**Supplementary Table S2.** Clinicopathological characteristics of TCGA-HNSCC cohort (Liu et al., Cell, 2018*)*.

**Supplementary Table S3.** The comprehensive list of the 1,020 identified proteins of TMT proteomics.

**Supplementary Table S4.** The antibody information used for Western Blot is shown in the table below.

| Antibody | Category | Working concentration |
| --- | --- | --- |
| CAV2 | NBP1-31116, Novus | 1: 1000 |
| PKR | 12297S, CST | 1: 1000 |
| PACT | 13490S, CST | 1: 1000 |
| Goat anti-Rabbit IgG | SA00001-2, Proteintech | 1: 10000 |
| Goat anti-Mouse IgG | SA00001-1, Proteintech | 1: 10000 |
| anti-Rabbit IgG | 30000-0-AP, Proteintech | 2µg for 1mg total protein lysate |
| P-eIF2α | 3398T, CST | 1: 1000 |
| Ubiquitin | 58395S, CST | 1: 1000 |
| GAPDH | 600004-1-Ig, Proteintech | 1: 50000 |
| β-Tublin | 2146S, CST | 1: 100 |

**Supplementary Table S5**. The primer sequences used for qRT–PCR analyses are shown in the table below.

| Gene Symbol | Forward Primers (5’-3’) | Reverse Primers (3’-5’) |
| --- | --- | --- |
| GAPDH | GTCTCCTCTGACTTCAACAGCG | ACCACCCTGTTGCTGTAGCCAA |
| β-actin | CACCATTGGCAATGAGCGGTTC | AGGTCTTTGCGGATGTCCACGT |
| CAV2 | TTCTCTTTGCCACCCTCAGCTG | GAAGCATCGTCCTACGCTCGTA |
| PKR | GAAGTGGACCTCTACGCTTTGG | TGATGCCATCCCGTAGGTCTGT |
| PACT | CCCTTAATGCCTGACCCTTCCA | CAGGAAGTCTCCAGCCATGATG |

**Supplementary File S1**: Full-length Western blot images are provided.
