# Supplementary material for: CAV2 Modulates Cetuximab Sensitivity in HNSCC via Ubiquitin-Mediated Disruption of the PACT-PKR Axis
Source: Cancers (Basel). 2026 Apr 2;18(7):1148. doi: 10.3390/cancers18071148 (PMC13072382; doi:10.3390/cancers18071148)

Figure 1B

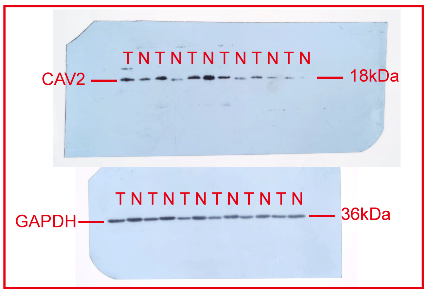

Figure 2A

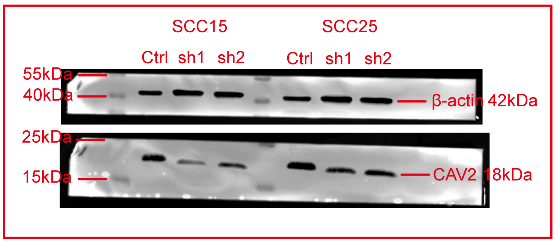

Figure 3A

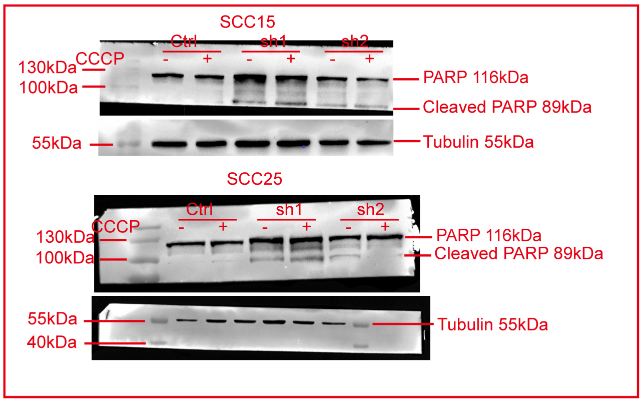

Figure 4C

SCC15 IP: PKR

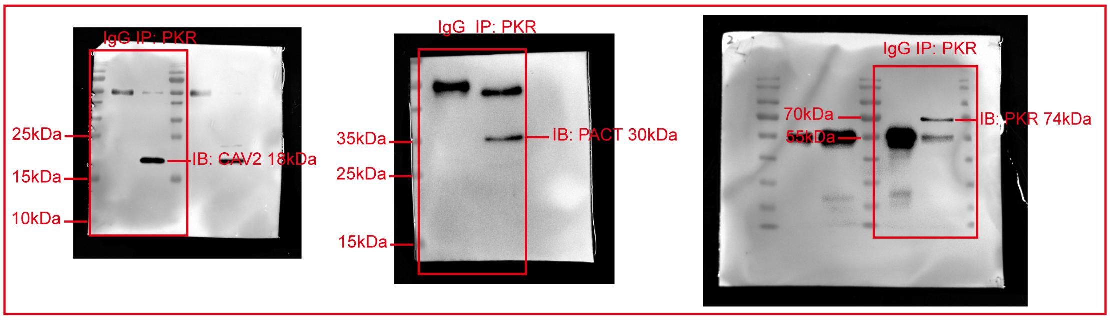

SCC15 IP: PACT

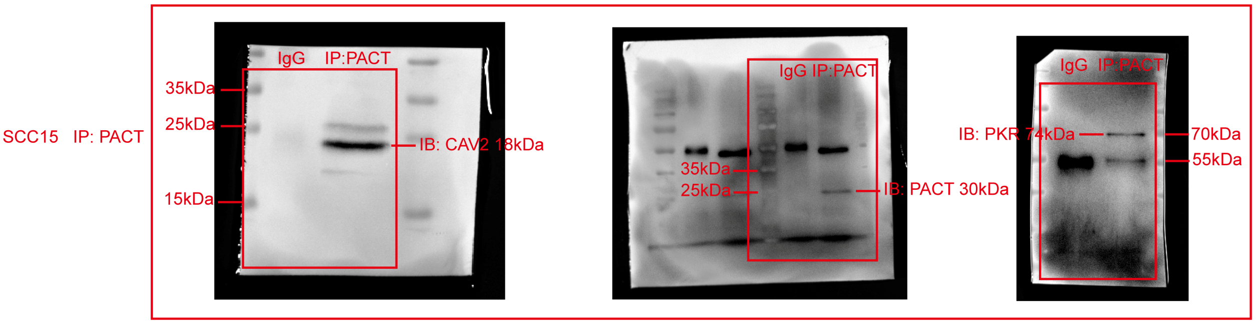

SCC15 IP: CAV2

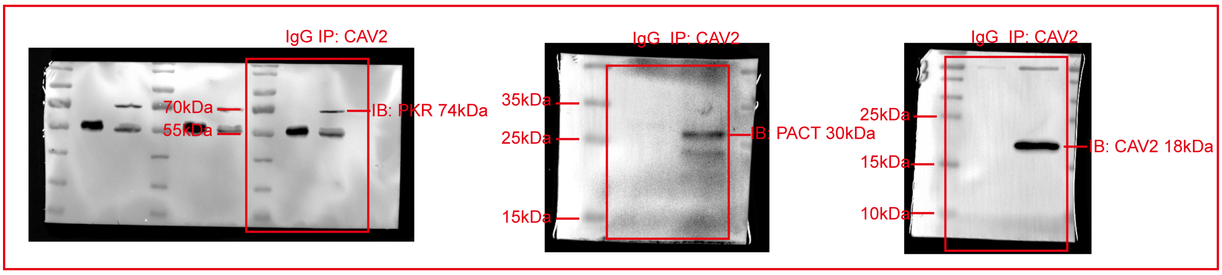

SCC15 Input

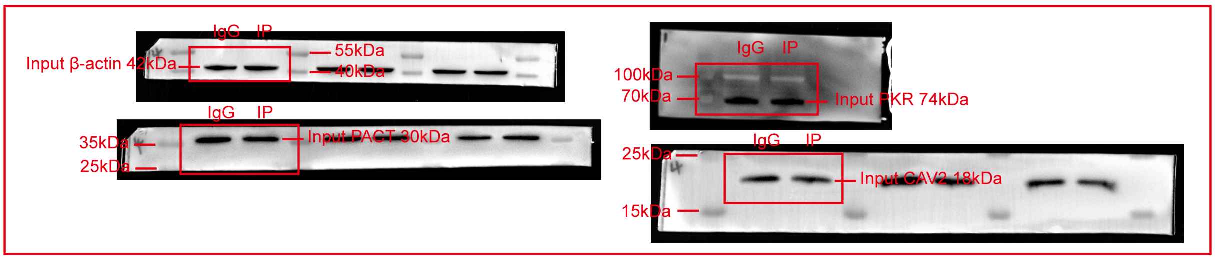

Figure 4C

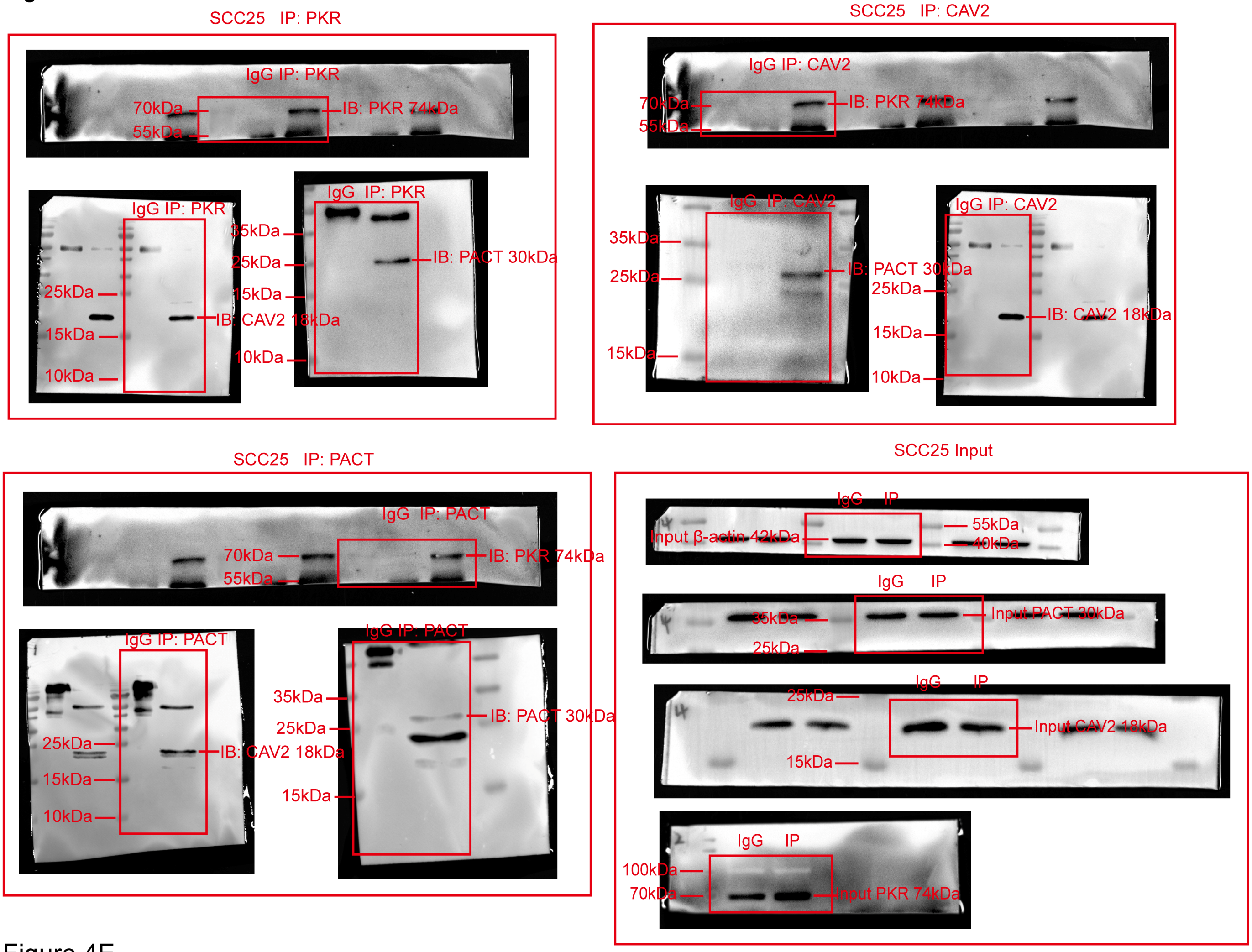

Figure 4E

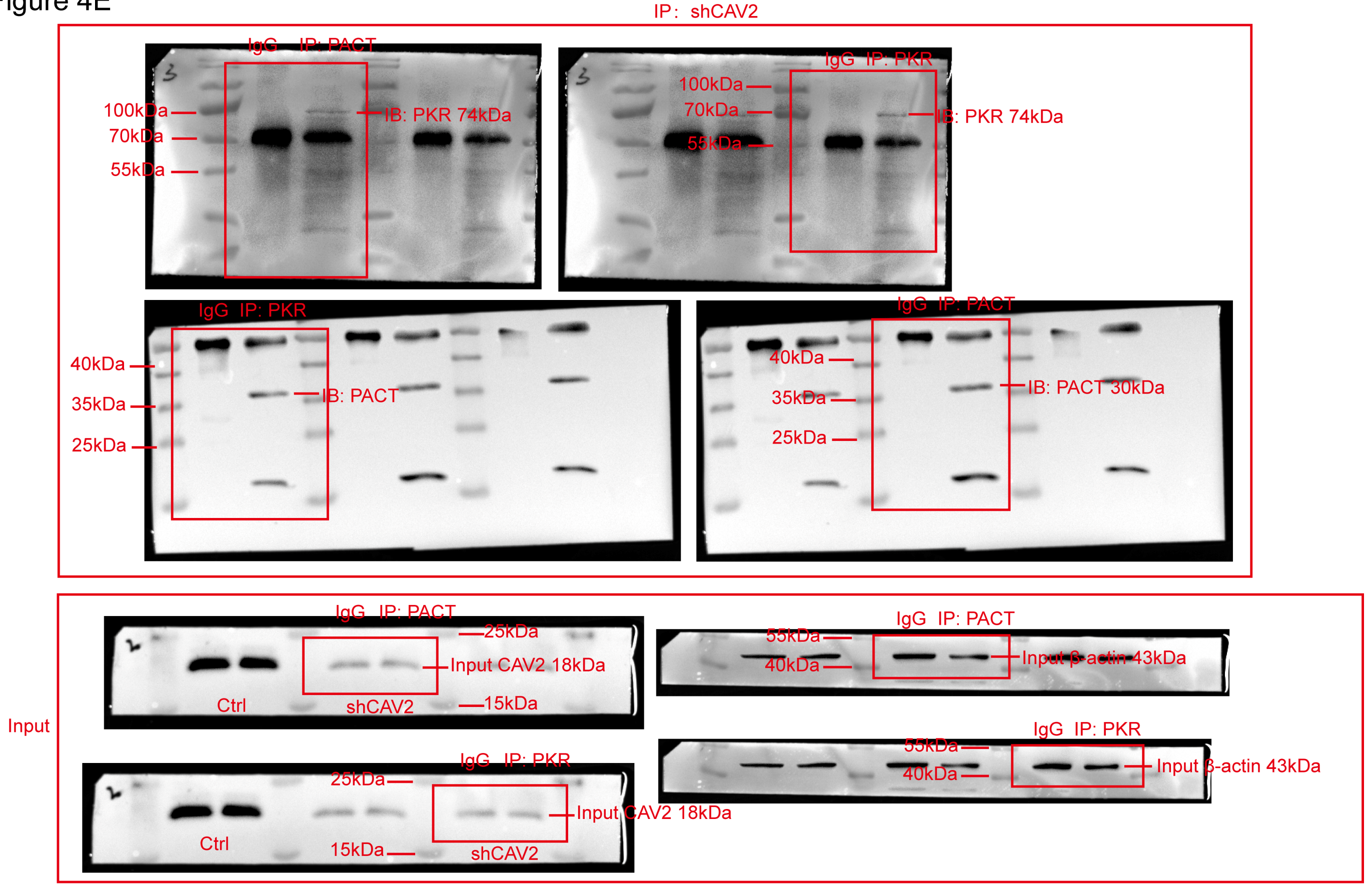

Figure 4F

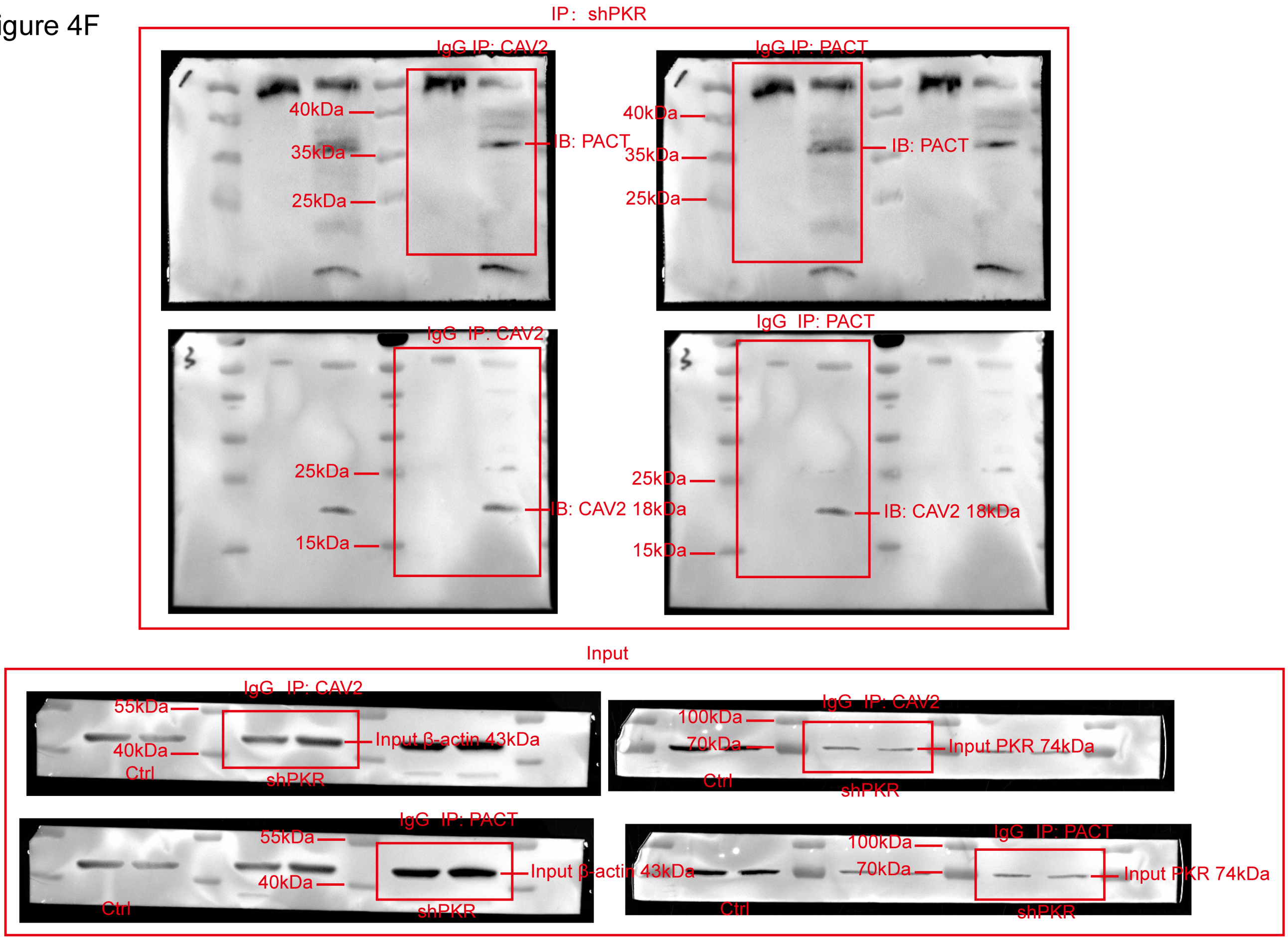

Figure 4G

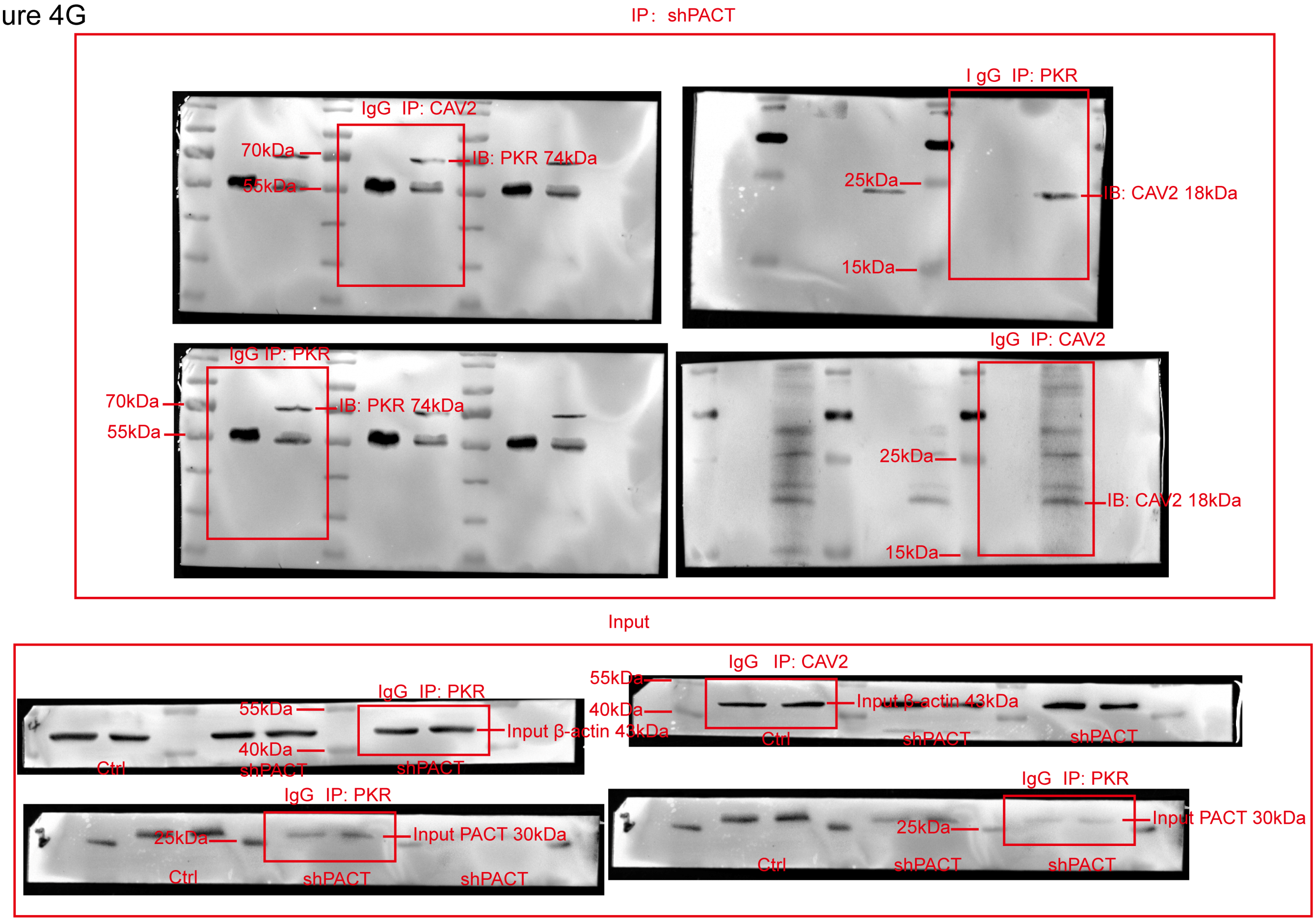

Figure 5A

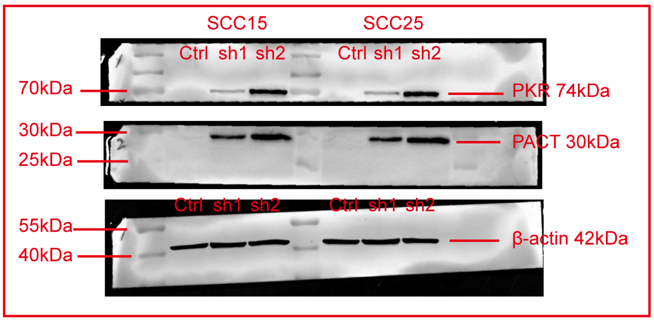

Figure 5B

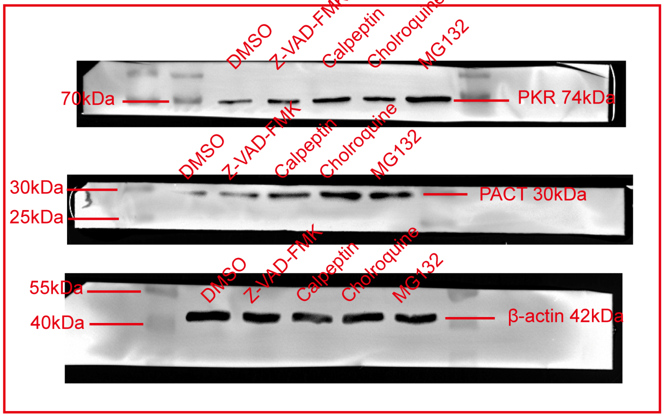

Figure 5C

SCC15 Ctrl

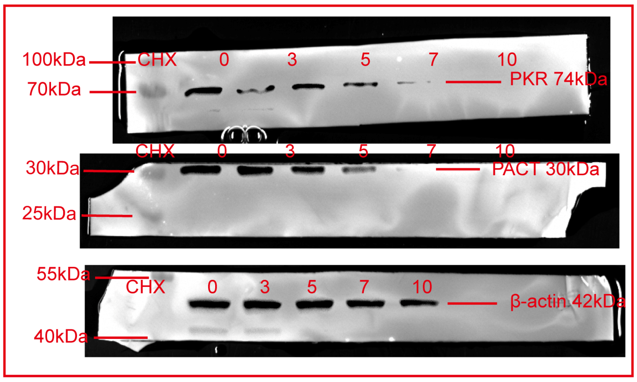

SCC15 shCAV2

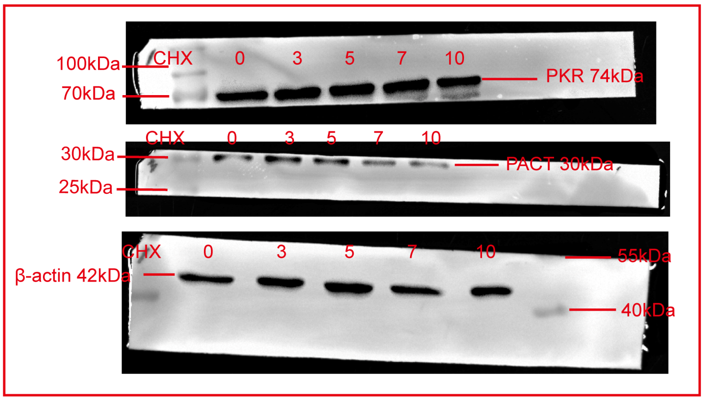

Figure 5D

SCC25 Ctrl

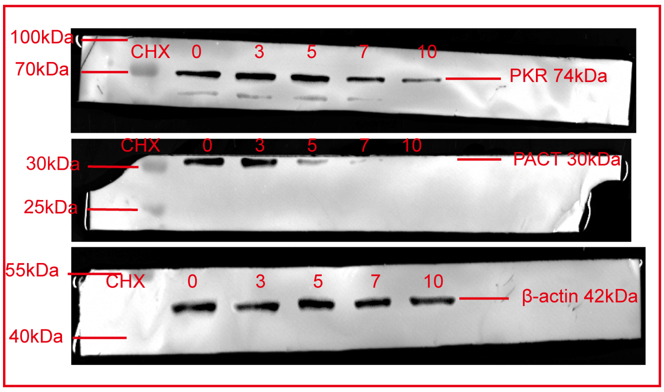

SCC25 shCAV2

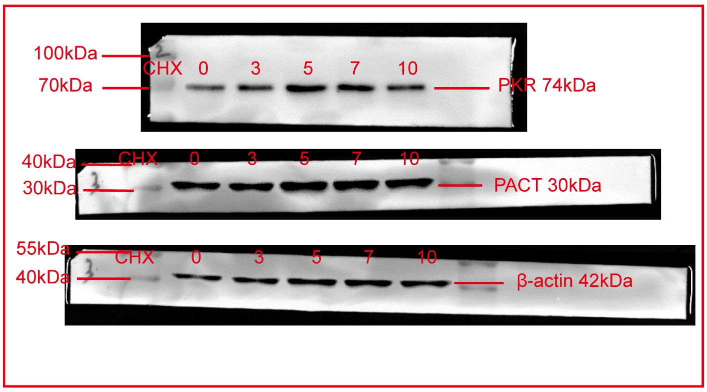

Figure 5E

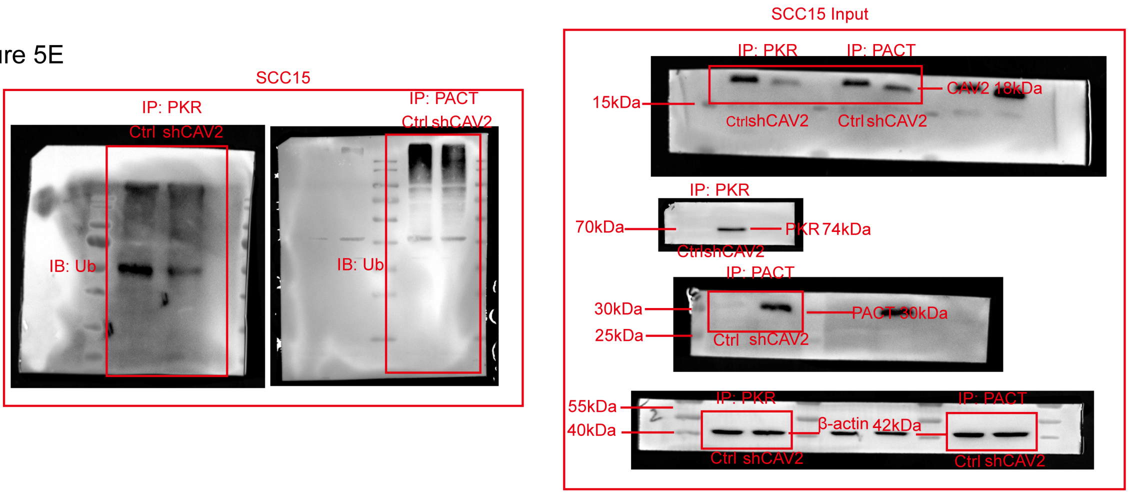

Figure 5F

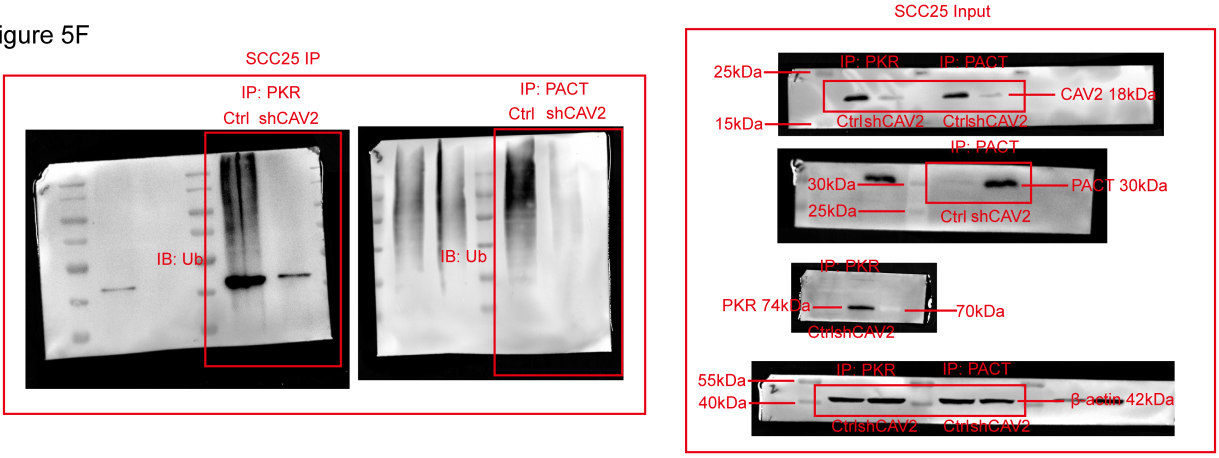

Figure 6A

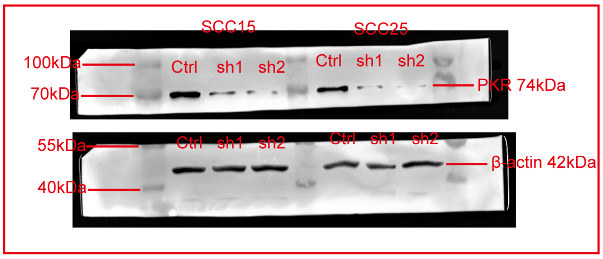

Figure 6C

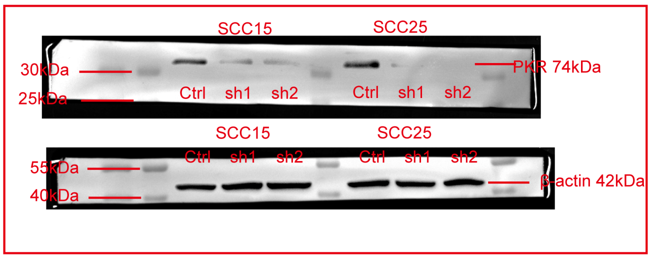

Supplementary Figure S3A

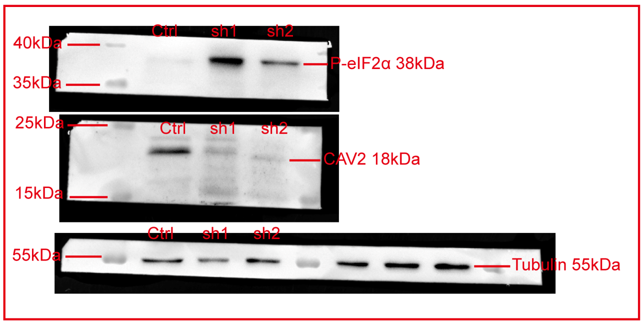

Supplement: Supplementary file 1 [file cancers-18-01148-s001.zip › Supplemenatry File S1 Original Western Data.pdf]
